# Dynamic Nutritional Trajectories in Growing–Finishing Pigs: A CRITIC-Based Evaluation and SHAP-Interpreted Final Body Weight Prediction

**DOI:** 10.3390/ani16213388

**Published:** 2026-10-27

**Authors:** Zhongshen Wang, Rui Sun, Jinsheng Li, Hailong Jiang

**Affiliations:** 1 College of Animal Science and Technology, Jilin Agricultural University, Changchun 130118, China; 2 Key Laboratory of Animal Production, Product Quality and Security, Ministry of Education, Changchun 130118, China; 3 Jilin Provincial Key Laboratory of Animal Nutrition and Feed Science, Changchun 130118, China; 4 College of Engineering and Technology, Jilin Agricultural University, Changchun 130118, China

**Keywords:** pig, growth and development, body conformation traits, nutritional regulation

## Abstract

Phase-specific nutrient supply influences growth performance, nutrient utilization, carcass development, and skeletal health. Dietary strategies are usually evaluated using a limited number of production traits. In this study, 165 pigs were assigned to nutritional trajectories across three body-weight phases: 25–50, 50–75, and 75–100 kg. Four dietary nutrient levels corresponding to 100%, 90%, 80%, and 70% of the reference nutrient concentrations were used. The trajectories included continuous nutrient supply, phase-specific nutrient restriction, nutrient switching, and nutritional recovery. A hierarchical indicator system was established using growth performance, nutrient digestibility, carcass traits, body conformation, energy and nitrogen deposition, bone geometry, trabecular microarchitecture, and bone mineralization traits. The Criteria Importance Through Intercriteria Correlation (CRITIC) method was used to determine indicator weights and calculate domain-specific and overall performance scores. Support vector regression, back-propagation neural network, and random forest models were developed to predict final body weight; SHAP analysis was used to interpret the contribution of input variables. Growth performance, energy and nitrogen deposition, and carcass quality received the highest objective weights. Combining internally validated predictions and model interpretation provides a quantitative basis for designing phase-specific nutritional strategies for growing–finishing pigs. Independent farm, breed, and management condition validation is required before practical application.

## Simple Summary

Designing precise feeding strategies to balance animal productivity, resource efficiency, and skeletal health is a central goal for sustainable pig production. Traditional evaluations of dietary nutrient levels often focus on a narrow set of growth traits, potentially overlooking the long-term impacts of dynamic nutrition on carcass quality and bone integrity. This study established a multi-dimensional evaluation framework by monitoring 165 growing–finishing pigs across three growth phases under various nutritional trajectories (ranging from 100% to 70% reference nutrient concentrations). By integrating comprehensive phenotypic data with machine learning models and the CRITIC weighting method, we found that nutritional levels during the middle-to-late stages are the primary drivers of final market weight. The results identified specific nutritional paths that optimize the balance between growth, nutrient utilization, and bone health. These findings provide a quantitative decision-making tool for precision phase-feeding management in the swine industry.

## 1. Introduction

The essence of the swine industry lies in the biological conversion and value addition of agricultural feedstuffs via swine metabolism [1]. Feed efficiency is fundamental to operational profitability and serves as a cornerstone for the sustainable development of the pig industry [2]. Currently, swine production in China faces unprecedented challenges, including volatile feed ingredient prices, increasingly stringent antibiotic reduction policies, and an urgent demand to mitigate environmental footprints, such as nitrogen and phosphorus emissions [3,4]. Given that feed costs consistently account for more than 60% of total production expenditure, marginal improvements in feed conversion efficiency can yield substantial economic and environmental benefits [5]. Consequently, moving beyond conventional extensive production models to enhance feed resource utilization and optimize nutrient efficiency has become imperative for improving productivity and efficiency across the industry.

Once digested and absorbed by pigs, dietary nutrients are systematically deposited in various tissues and organs. The nutrient requirements of pigs encompass three distinct dimensions: maintenance of metabolism, growth and development, and immune regulation. The development of tissues and organs follows a strict chronological sequence; across the entire production cycle from weanling to finishing pigs, the digestive tract, skeleton, muscle, and adipose tissues reach their developmental peaks sequentially. Owing to the differences in nutrient composition and accretion rates among different tissues and organs [6], swine dietary nutrient requirements, both quantitatively and qualitatively, exhibit pronounced spatiotemporal dynamics throughout the growth trajectory.

In traditional swine husbandry, empirical practices such as “frame-building” (deliberately restricting dietary nutrient density during the growing stage to foster a larger skeletal frame, causing compensatory fat growth) have been widely used [7]. However, when applied to modern, high-performing genetic lines, this empirical regime faces numerous unresolved scientific questions under the constraints of modern genetics, feed conversion rates, and herd turnover. For instance, the precise regulation of skeletal development by dietary energy and protein levels, the synergistic mechanisms through which genetic background governs muscle and fat accretion, and whether nitrogen deposition efficiency is strongly associated with skeletal growth are unclear.

Phase-restricted feeding strategies, often dynamically adjusted in commercial operations according to market fluctuations, demand a paradigm shift from empirical to science-based precision nutrition programs. Leveraging modern precision nutrition theory and data science to systematically assess multidimensional phenotypes (including feed conversion efficiency, body tissue development, nitrogen retention efficiency, and carcass traits) has emerged as a mainstream trajectory toward data-driven swine herd management [8,9]. Using data cleaning and dimensionality reduction across multisource production datasets, robust nutrient requirement prediction models can be established across distinct physiological stages. Precisely matching dietary nutrient density with the biological rhythms of tissue development will not only optimize the full genetic growth potential of pigs, but also effectively minimize nutrient overexcretion, thereby reducing the environmental burden.

Historically, evaluation paradigms have disproportionately focused on isolated growth performance traits and have failed to capture the systemic effects of dietary interventions on swine physiological metabolism and overarching production benefits. To extract critical information from intricate, multidimensional production datasets, a scientifically rigorous, comprehensive evaluation system is required. Objective weighting methods are preferable to subjective approaches because they eliminate human bias. However, when evaluating multi-tiered indicators encompassing growth performance, carcass traits, and skeletal development, severe multicollinearity frequently arises. Conventional objective weighting techniques, such as the entropy weight method, often result in distorted weight assignments because of the redundant counting of highly correlated information sources [10]. To overcome this limitation, in the present study, we introduce the Criteria Importance Through Intercriteria Correlation (CRITIC) method [11]. This method not only measures the intrinsic information contrast of each indicator via standard deviation but also incorporates a conflict index based on correlation coefficients to identify redundant information. In multi-tiered, highly collinear indicator systems, the CRITIC method effectively suppresses the weight inflation caused by multicollinearity, ensuring that the true informative value of each metric is objectively reflected. Accordingly, we used this data-driven analytical tool to objectively calculate trait weights across multiple phenotypic tiers, thereby constructing and optimizing a comprehensive nutritional trajectory evaluation framework and a final body weight (FBW) prediction model. By mining core predictive and evaluative indicators, we aimed to provide a solid theoretical basis and empirical data support for the formulation and execution of precise nutrition strategies in growing–finishing pigs, ultimately contributing to a more efficient, resilient, and sustainable future for the swine industry.

## 2. Materials and Methods

### 2.1. Ethics Statement

All animal procedures were conducted in accordance with the Guidelines for the Care and Use of Laboratory Animals established by Jilin Agricultural University. The experimental protocols were reviewed and approved by the Ethics Committee of Jilin Agricultural University (Approval No.: 2025073002). The animal trial was conducted from 2 August 2025 to 25 December 2025.

### 2.2. Experimental Design and Animals

To capture experimental datasets spanning the targeted growth trajectory, a total of 165 healthy Duroc × Landrace × Yorkshire (DLY) crossbred, growing pigs (average age: 60 d; initial body weight [BW]: 22.9 ± 3.2 kg) were selected for this study (all pigs used in this study were barrows). A 10-day adaptation period was provided prior to the formal trial to acclimatize the pigs to the FIRE electronic feeding system. The feeding trial was divided into three distinct growth phases: Phase 1 (25–50 kg BW), Phase 2 (50–75 kg BW), and Phase 3 (75–100 kg BW). A standard corn–soybean meal basal diet was formulated to serve as the 100% nutrient density diet (Diet A). Based on Diet A, three test diets with reduced energy and nutrient densities were prepared by proportionally diluting the nutrient concentrations to 90% (Diet B), 80% (Diet C), and 70% (Diet D) of the basal level.

**Figure 1:**
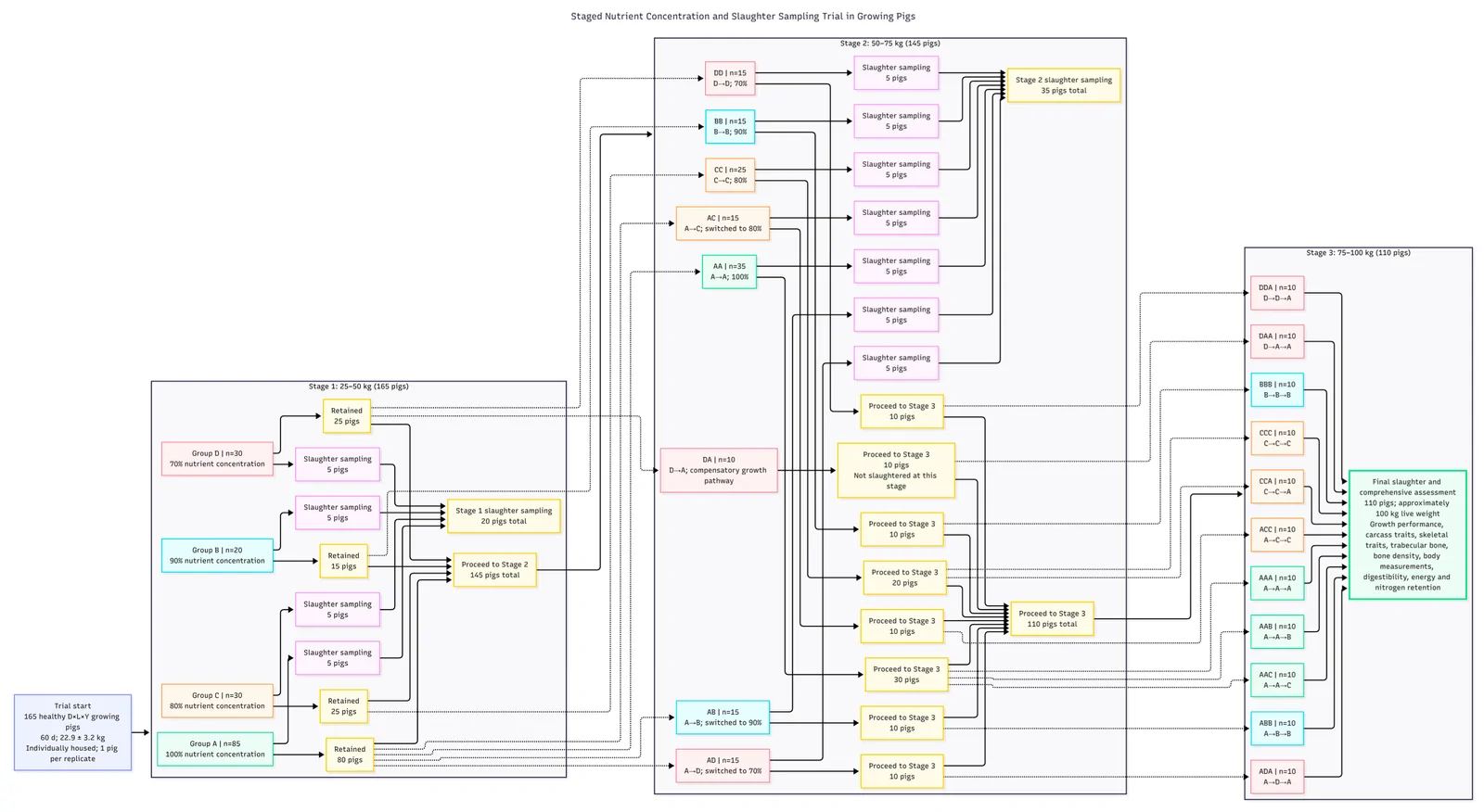
Schematic diagram of the experimental design and group allocation of the pigs.

A dynamic nutritional trajectory design was implemented to evaluate the biological effects of sustained nutrient supply, step-down nutrient dilution, and compensatory growth-feeding strategies. A detailed allocation flowchart is shown in Figure 1.

Phase 1 (25–50 kg): Based on initial BW, the pigs were randomly allocated to four dietary treatment groups: Group A (Control; fed Diet A [100% nutrient density]; *n* = 85), Group B (fed Diet B [90% nutrient density]; *n* = 20), Group C (fed Diet C [80% nutrient density]; *n* = 30), and Group D (fed Diet D [70% nutrient density]; *n* = 30). At the conclusion of Phase 1, five pigs from each group were randomly selected and slaughtered to evaluate carcass traits and skeletal characteristics. The remaining 145 pigs advanced to the subsequent phase.

Phase 2 (50–75 kg): The remaining 145 pigs were reallocated to eight treatment groups to model nutritional trajectory shifts. Treatments were designated by combining the letter codes representing the diets fed in each phase (e.g., “AB” denotes pigs fed Diet A in Phase 1 and switched to Diet B in Phase 2).

Continuous Trajectories: Groups AA (*n* = 35), BB (*n* = 15), CC (*n* = 25), and DD (*n* = 15) maintained their original dietary regimens (Diets A, B, C, and D, respectively).

Step-Down Trajectories: Groups AB (*n* = 15), AC (*n* = 15), and AD (*n* = 15) were randomly subsampled from the original Group A and switched to Diets B, C, and D, respectively.

Compensatory Trajectory: Group DA (*n* = 10) was subsampled from Group D and switched to Diet A to evaluate the compensatory growth response following nutritional rehabilitation from a low-density regimen to the control level (Group DA was evaluated exclusively for growth performance; no slaughtering was conducted in this group at the end of this phase). At the end of Phase 2, five pigs were randomly selected for slaughter and sample collection from all groups except Group DA. The 110 remaining pigs entered in the final phase of the trial.

Phase 3 (75–100 kg): The remaining 110 pigs were divided into 11 final trajectory groups (*n* = 10 per group). A three-letter nomenclature was used to denote the complete chronological feeding trajectory across all three phases:

Continuous Trajectories: Groups AAA (positive control), BBB, and CCC continued to receive Diets A, B, and C, respectively.

Step-down Trajectories: Groups AAB and AAC were sub-sampled from Group AA and transitioned to Diets B and C, respectively; Groups ABB, ACC, and ADA represented the continuation of treatments from Groups AB, AC, and AD, respectively.

Compensatory Trajectories: Group CCA was sub-sampled from Group CC and switched to Diet A; Group DDA maintained the DD treatment and was subsequently transitioned to Diet A; Group DAA served as the continuation of the compensatory regimen from Group DA in Phase 2. When the pigs in each of the 11 groups reached the target average BW of approximately 100 kg, five representative pigs were randomly selected from the ten available animals per group for terminal slaughter and comprehensive biological sampling. Meanwhile, the remaining five pigs in each group continued to be fed and monitored for growth performance until they reached approximately 130 kg. This design was deliberately implemented to provide an extended dynamic range of phenotypic data for the subsequent machine learning growth models, ensuring robustness in final body weight predictions. The measured parameters included growth performance, carcass traits and quality, skeletal and trabecular microarchitectural characteristics, bone mineral density (BMD), morphometric body measurements, nutrient digestibility, and energy and nitrogen retention.

### 2.3. Experimental Diets and Feeding Management

Conventional and unconventional feed ingredients, including corn, soybean meal, wheat middling, and wheat bran, were used in this study. These ingredients were selected based on their nutrient profiles and potential utilization in swine diets, and their nutritional compositions and physical characteristics were analyzed (Table 1). The procedures for determining the chemical composition and physical properties of the feed ingredients and diets adhered to the methodologies described previously [12]. The detailed chemical composition, nutrient profiles, and physical characteristics of the individual feed ingredients are presented in Table 1, and the ingredient composition and calculated/analyzed nutrient levels of the experimental diets are presented in Table 2. The basal diet (100% nutrient density, Diet A) was formulated to meet or exceed the nutritional requirements recommended by the National Research Council (NRC, 2012) [13] for growing–finishing pigs. To establish the dynamic nutritional trajectories, the diluted diets (i.e., 90%, 80%, and 70% nutrient densities) were subsequently formulated proportionally based on the basal diet to achieve their respective targeted nutritional levels. The experimental diets were formulated by incorporating the corncob diluent at 10%, 20%, and 30%, which resulted in graded restrictions of the basal nutrient intake. To achieve the precise dilution of dietary nutrient densities, corncobs were mechanically milled (with the inner pith removed) and finely ground into a powder. Following toxicological screening and in vitro digestibility assays, the ground corncob powder was incorporated into the basal diet as a low-nutrient fibrous diluent [14]. The feeding trial was conducted at the Swine Experimental Base of the Jilin Academy of Agricultural Sciences. Feeding was managed using an automated Feed Intake Recording Equipment (FIRE) feeding station system (Osborne Industries Inc., Osborne, KS, USA). Each pig was fitted with a radio-frequency identification (RFID) ear tag, enabling real-time monitoring of daily individual body weight, feed consumption, duration of visits, and feeding frequency. Every 5 days throughout the trial, the individual weight scales and electronic feed hoppers of the FIRE stations were calibrated using certified standard weights (50 kg, 20 kg, and 10 kg for the weight scales; 1 kg and 500 g for the feed hoppers), with structural leveling checks. The health status of the pigs and the operational integrity of the FIRE system were monitored daily. Facility disinfection and baseline vaccination regimens adhered strictly to the farm’s standard operating procedures. The ambient temperature, relative humidity, and indoor air quality were regulated using combined natural and mechanical ventilation. Slatted floors and dung alleys were cleaned daily in the afternoon, and manure was collected using an automated scraper system to maintain pen hygiene. A total of 165 pigs were allocated across 12 identical pens within a single environmentally controlled room. The pen density was maintained at no more than 15 pigs per pen. Each pen was equipped with one Osborne Feed Intake Recording Equipment (FIRE) station, which dispensed one specific diet. The FIRE system is a single-space electronic feeder that allows only one pig to enter and isolate itself during feeding, thereby accurately capturing independent individual feed intake and body weight data while minimizing feeding competition. A 10-day adaptation period was provided prior to the formal trial to acclimatize the pigs to the FIRE electronic feeding system. Throughout the experimental period, all the pigs had ad libitum access to food and fresh water. The pen density was maintained at no more than 15 pigs per pen.

### 2.4. Energy Metabolism Trial

An energy metabolism trial was conducted using open-circuit indirect respiration calorimetry chambers at the Jilin Academy of Agricultural Sciences. Prior to chamber entry, pigs in each group were individually housed in metabolic pens for a 14-day acclimation period to adapt to the experimental diets and housing conditions. From day 15 onward, pigs in Phase 1 were transferred into the respiration chambers (*n* = 12, with three pigs per dietary treatment group; 7-day duration). Phase 2 evaluations commenced on day 35 and involved two consecutive batches (four groups per batch, 12 pigs per batch, three pigs per treatment; 7-day duration per batch). Phase 3 evaluations commenced on day 85 and were conducted across three consecutive batches. Batches 1 and 2 each evaluated four groups (12 pigs per batch, resulting in a combined total of 24 pigs); Batch 3 evaluated the remaining three groups (a total of nine pigs). Across all batches, there were exactly three pigs evaluated per treatment group. For the respiration chamber experiments, the selected pigs were those whose body weights were closest to the average body weight of their respective replicate pens.

During each respiration trial, the pigs were fed their respective experimental diets at a daily intake level set at three times the metabolizable energy requirement for maintenance (419 kJ/kg BW^0.75d^) [15,16]. Consequently, the physical daily feed allowance (kg as-fed) was proportionally adjusted according to the varying energy densities of the diluted diets. Pigs were weighed every 7 days to adjust the daily feed allowance accordingly. Daily oxygen consumption (O_2_) and carbon dioxide (CO_2_) and methane (CH_4_) emissions were continuously recorded. Following chamber entry, total fecal and urinary samples were collected from days 2–6 from each batch, concomitant with the recording of respiratory gas exchange to quantify total heat production (HP). Fasting heat production (FHP) was determined to evaluate basal fasting metabolic levels. The fasting heat production (FHP) was experimentally measured and calculated using the same equation applied for total heat production (THP). The FHP calculation utilized gas concentrations and air flow data obtained specifically from the last 8-h heat production measurement window on day 16 (i.e., from 22:30 to 06:30 h, starting 31 h after the last maintenance meal). To standardize the production metric over the same time span used for THP, this 8-h heat production measurement was extrapolated to a 24-h period [17]. All pigs had ad libitum access to fresh water. Daily feed allowances were partitioned into two equal meals offered at 08:00 and 16:00. Total feces and urine were collected each morning, weighed, and stored at −20 °C. (Note on excluded period: gas exchange recorded during the acclimation phase was excluded from subsequent computational analyses). Following completion of the trial, pooled fecal and urine subsamples from each pig were thoroughly homogenized and processed for subsequent proximate nutrient analysis.

Digestible energy (DE), ME, THP, heat increment (HI), RE, NE, CH4 energy, and respiratory quotient (RQ) were calculated according to previous reports [18,19]:

DE (MJ/d) = GE_intake_ (MJ/d) − fecal_energy_ (FE,MJ/d) (1)

ME (MJ/d) = DE (MJ/d) − urinary energy (UE,MJ/d) − CH_4_ energy (MJ/d) (2)

THP (kJ) = 16.181 O_2_ consumption (L) + 5.023 CO_2_ excretion (L) − **2.168 CH_4_ excretion (L) − 5.989 urinary nitrogen (UN,g)** (3)

HI (MJ/d) = THP (MJ/d) – FHP (MJ/d)(4)

RE (MJ/d) = ME (MJ/d) – THP (MJ/d)(5)

NE (MJ/d) = RE (MJ/d) + FHP (MJ/d)(6)

(7) $$\mathrm{RQ}=CO_{2}\mathrm{excretion}\left(L\right)/O_{2}\mathrm{consumption}\left(L\right)$$

CH_4_ energy (kJ/d) = CH_4_ (L/d) × 39.6 (kJ/L) (8)

### 2.5. Growth Performance

During the trial period, individual body weight and feed intake data were automatically recorded using the FIRE system. Pig body weights were recorded at the beginning and end of each phase. Feed intake during the formal trial period was based on individual records from the FIRE system, following outlier screening and manual verification.

Average daily gain (ADG), average daily feed intake (ADFI), and feed conversion ratio (FCR) were calculated using the following equations:

(9) $$\mathrm{ADG}=\frac{W_{\mathrm{end}}-W_{\mathrm{start}}}{T}$$

(10) $$\mathrm{ADFI}=\frac{F_{\mathrm{total}}}{T}$$

(11) $$\mathrm{FCR}=\frac{\mathrm{ADFI}}{\mathrm{ADG}}$$

where W_end_ is the body weight at the end of the phase and W_start_ is the body weight at the beginning of the phase, expressed in kg; F_total_ is the total feed intake during the formal trial phase, expressed in kg; and T is the number of formal trial days, expressed in d. The units for ADG and ADFI are kg/d, whereas FCR is a dimensionless indicator.

Raw data from the FIRE system were first subjected to individual identity matching, time continuity checks, body weight rationality checks, and feed record outlier screening. Data generated from equipment malfunction, ear tag identification failure, obvious feed spillage, or abnormal feeding behavior were excluded based on predefined quality control rules, and a data cleaning log was maintained. Selective deletion based solely on treatment group differences was strictly prohibited.

The compensatory growth index (CGI) was used to describe the extent of body weight recovery in restricted animals after nutritional rehabilitation, calculated as follows:

(12) $$\mathrm{CGI}=\frac{A-B}{A}\times 100\%$$

where A is the body weight difference between normal-nutrition animals and restricted-nutrition animals at the end of the restriction period, and B is the body weight difference between the normal-nutrition animals and the previously restricted compensatory animals at the end of the compensatory growth period. This indicator was only used to describe the degree of compensatory recovery and did not serve as an independent primary statistical endpoint; formal inferences remained based on individual-level repeated measurement data and mixed-effects models.

### 2.6. Nutrient Digestibility

Apparent digestibility was determined using the total feces collection method. As previously described, total feces produced by each individual pig in the respiratory calorimeter were completely collected and weighed every morning from day 2 to 6 of each batch. The level of replication for the digestibility calculation was the individual pig (*n* = 6 per treatment). To prevent nitrogen loss, 10% sulfuric acid was added to the daily collected feces for nitrogen fixation. At the end of the 5-day collection period, the feces collected from each individual pig were thoroughly pooled, and a representative sub-sample was taken and stored at −20 °C for further nutrient determination. Feed and fecal samples were dried in a 65 °C oven for 12 h and 72 h, respectively. The dried samples were equilibrated overnight at room temperature, ground, passed through a 40-mesh sieve, and stored in sealed containers until analysis. According to the “Feed Analysis and Feed Quality Testing Technology” and relevant national standards, the dry matter, crude protein, crude fat, ash, and crude fiber contents in feed and feces were determined. Crude protein was determined using an automatic Kjeldahl nitrogen analyzer, ash by the muffle furnace ashing method, crude fat by the Soxhlet extraction method, and gross energy by an oxygen bomb calorimeter.

In cases where complete fecal collection was performed without the use of exogenous indicators, the apparent digestibility of each nutrient was calculated as follows:

(13) $$D_{\mathrm{nutrient}}=\frac{I_{\mathrm{nutrient}}-E_{\mathrm{nutrient}}}{I_{\mathrm{nutrient}}}\times 100\%$$

where *I_nutrient_* is the intake of the corresponding nutrient during the trial, and *E_nutrient_* is the fecal output of the corresponding nutrient. Specific calculations are as follows:

(14) $$D_{\mathrm{CF}}=\frac{I_{\mathrm{CF}}-E_{\mathrm{CF}}}{I_{\mathrm{CF}}}\times 100\%$$

(15) $$D_{\mathrm{EE}}=\frac{I_{\mathrm{EE}}-E_{\mathrm{EE}}}{I_{\mathrm{EE}}}\times 100\%$$

(16) $$D_{\mathrm{CP}}=\frac{I_{\mathrm{CP}}-E_{\mathrm{CP}}}{I_{\mathrm{CP}}}\times 100\%$$

(17) $$D_{\mathrm{GE}}=\frac{I_{\mathrm{GE}}-E_{\mathrm{GE}}}{I_{\mathrm{GE}}}\times 100\%$$

where *D_CF_*, *D_EE_*, *D_CP_* tand *D_GE_* represent the apparent digestibility of crude fiber, crude fat, crude protein, and gross energy, respectively.

Nutrient digestibility indicators used for the CRITIC comprehensive evaluation included crude fiber digestibility, crude fat digestibility, crude protein digestibility, and energy digestibility.

### 2.7. Carcass Quality

Carcass traits were determined according to the method detailed in [20,21]. Measured indicators included dressing percentage, leaf fat weight, loin muscle area, backfat thickness, straight carcass length, oblique carcass length, and skin thickness. Pigs were weighed prior to slaughter to obtain live weight. After slaughter and removal of blood, viscera, head, hooves, and other specified parts, the carcass was weighed. Dressing percentage was calculated as follows:

(18) $$\mathrm{Dressing percentage}=\frac{W_{\mathrm{carcass}}}{W_{\mathrm{live}}}\times 100\%$$

where W_carcass_ the carcass weight, and W_live_ is the pre-slaughter live weight.

Loin muscle area (LMA) was determined by measuring the cross-sectional area of the longissimus dorsi muscle at the last rib and calculated as follows:

LMA = H × W × 0.7(19)

where H is the height of the loin muscle, and W is the width of the loin muscle.

Backfat thickness was determined on the right half-carcass in a suspended state by averaging measurements taken at the thickest part of the midline, the last rib, and the lumbo-sacral junction. Straight carcass length was the straight-line distance from the anterior edge of the pubic symphysis to the anterior edge of the first cervical vertebra; oblique carcass length was the diagonal distance from the anterior edge of the pubic symphysis to the junction of the first rib and the sternum. Skin thickness was measured at the junction of the 6th and 7th thoracic vertebrae. All length and thickness indicators were measured using calibrated measuring tapes or vernier calipers [22,23].

Carcass indicators used for the comprehensive evaluation included dressing percentage, straight carcass length, oblique carcass length, loin muscle area, backfat thickness, and skin thickness. Backfat thickness was temporarily treated as a positive indicator in the CRITIC comprehensive evaluation, reflecting that under the production evaluation framework set in this study, moderate fat deposition was considered a component of carcass development and commercial assessment. To prevent interpretation bias from this assumption, a sensitivity analysis was conducted by recalculating the comprehensive score with backfat thickness set as both a positive and a negative indicator, and its impact on the ranking results was evaluated.

### 2.8. Bone Sample Collection and Micro-CT Analysis

At the end of phases 1, 2, and 3, 5 pigs from each treatment group were randomly selected for slaughter and bone sample collection. Pigs were humanely slaughtered in accordance with GB/T 22569-2008 [24] “Technical Specification for Humane Slaughter of Pigs”. The right hind limb was taken, sectioned at the fibula–tarsus and tibia–tarsus junctions to separate the hoof, and after removing muscle, fascia, and other attached tissues, the fourth metatarsal bone was collected as the test sample. Samples were immediately fixed in 4% paraformaldehyde and stored at 4 °C until Micro-CT analysis.

**Figure 2:**
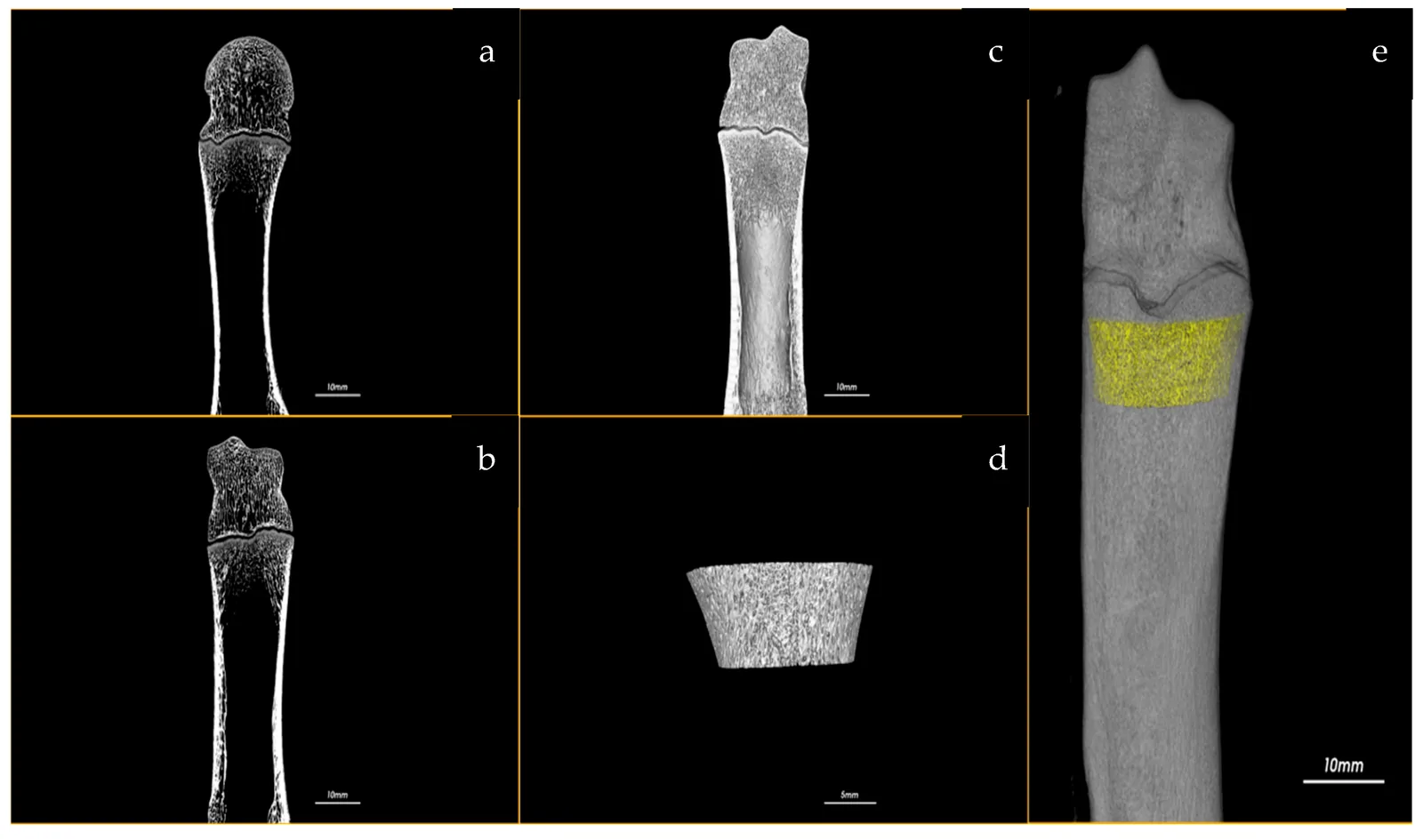
Scanned images of metatarsal samples. (**a**) 2D coronal plane; (**b**) 2D sagittal plane; (**c**) 3D cross-section; (**d**) 3D trabecular bone; (**e**) region of interest (ROI).

A Micro-CT scanner (NMC-200, Pingsheng Healthcare, Kunshan, China) was used to scan the in vitro bone samples. Raw images obtained from scanning were acquired via Cruiser 2.3 software, subjected to three-dimensional reconstruction using Recon 1.7.0 software, and analyzed in the target region of interest (ROI) using Avatar 1.7.7 software (Figure 2). TMicro-CT scanning and subsequent data analysis of the fourth metatarsal were performed by a specialized commercial imaging facility. The region of interest (ROI) was consistently positioned in the proximal metaphysis across all samples. Specifically, the ROI started immediately distal to the proximal growth plate and extended approximately 10 mm distally along the longitudinal axis. Standard manufacturer-supplied calibration phantoms and internal quality control protocols were strictly utilized by the facility to ensure the accuracy of BMD/TMD calibration and overall measurement consistency.

Bone parameters included: bone volume, BV; tissue volume, TV; bone volume fraction, BV/TV; bone surface area, BS; bone surface to tissue volume ratio, BS/TV; bone surface to bone volume ratio, BS/BV.

Trabecular parameters included: trabecular thickness, Tb. Th; trabecular separation, Tb. Sp; trabecular number, Tb. N; trabecular pattern factor, Tb. Pf; structure model index, SMI.

Bone density and mineral parameters included: bone mineral density, BMD; bBone mineral content, BMC; tissue mineral density, TMD; tissue mineral content, TMC (Table 3).

### 2.9. Body Measurement Traits

During the trial period, manual measurements of body dimension traits were taken every 5 d using measuring sticks, tape measures, and angle rulers to calibrate the FIRE system’s body weight data and to describe the physical development of the pigs. Body weight was adopted from the individual weight settled by the FIRE system on the day prior to the measurement day. Prior to measurement, pigs were made to stand naturally with weight evenly distributed across all four limbs and the head kept in a natural posture. The measurement indicators are defined as follows:

Body length (BL): the distance from the midpoint of the line connecting the bases of the ears to the recess of the tail base; chest width (CW): the transverse straight-line distance between the left and right sides of the chest; chest height (CH): the vertical distance from the highest projection point of the chest width line on the back to the ground; rump width (RW): the width between the outer edges of the coxal tubers on both sides; rump height (RH): the vertical distance from the highest projection point of the rump width line on the back to the ground.

### 2.10. Selection of Comprehensive Evaluation Indicators

In this study, the CRITIC method was employed to comprehensively evaluate the multidimensional phenotypes of pigs subjected to different nutritional trajectories. Because the CRITIC weighting method simultaneously accounts for the contrast intensity of indicators and the conflict between them, it requires that the selected indicators possess relatively complete datasets, explicit biological directionality, and relatively independent informational contributions [25,26].

The final indicators incorporated into the evaluation system, along with their assigned directionalities, are detailed in Table 4.

Backfat thickness was treated as a positive indicator, as excessive subcutaneous fat deposition is generally undesirable in modern lean-type pig production.

Tissue Volume (TV) of the bone was defined as a positive indicator, as a larger TV reflects a more robust macro-geometric expansion of the skeletal frame during the rapid growth phase.

Bone Surface/Bone Volume (BS/BV): While often inversely related to Trabecular Thickness (Tb. Th) in aging models, a higher BS/BV in rapidly growing young pigs indicates a larger active surface area available for dynamic bone modeling and osteoblast activity. Thus, it was considered a positive indicator of active skeletal development in our specific physiological context.

### 2.11. CRITIC Comprehensive Evaluation Method

The CRITIC (Criteria Importance Through Intercriteria Correlation) method was employed to comprehensively evaluate the multi-dimensional phenotypes of pigs under different nutritional trajectories. This method simultaneously accounts for the contrast intensity of indicators and the conflict between them. A higher standard deviation indicates a stronger discriminative ability, while a lower correlation with other indicators signifies a greater amount of independent information, resulting in a higher objective weight. Furthermore, the CRITIC method intrinsically accounts for highly correlated or overlapping indicators across domains by mathematically down-weighting redundant information based on their intercriteria correlation. Let there be n evaluation objects and m evaluation indicators, forming the original data matrix:

(20) $$X={\left(x_{\mathrm{ij}}\right)}_{n\times m}$$

where *x_ij_* represents the observed value of the i-th object for the j-th indicator. Based on biological significance, indicators were categorized as positive or negative and subjected to dimensionless processing using the range method. Positive indicators were standardized as:

(21) $$z_{\mathrm{ij}}=\frac{x_{\mathrm{ij}}-x_{j,\min}}{x_{j,\max}-x_{j,\min}}$$

Negative indicators were standardized as:

(22) $$z_{\mathrm{ij}}=\frac{x_{j,\max}-x_{\mathrm{ij}}}{x_{j,\max}-x_{j,\min}}$$

where *z_ij_* is the standardized value, and *x_j_*_,*max*_ and *x_j_*_,*min*_ are the maximum and minimum values of the *j*-th indicator, respectively. Indicators with no discriminative ability (where maximum equals minimum) were excluded from weight calculation.

The contrast intensity of the *j*-th indicator was expressed by the sample standard deviation

(23) $$S_{j}=\sqrt{\frac{\sum_{i=1}^{n}{\left(z_{\mathrm{ij}}-{\bar{z}}_{j}\right)}^{2}}{n-1}}$$

where ${\bar{z}}_{j}$ is the mean of the standardized values for the j-th indicator. The Pearson correlation coefficient was used to evaluate the degree of conflict, calculated as:

(24) $$R_{j}=\sum_{k=1}^{m}\left(1-r_{\mathrm{jk}}\right)$$

where *r_jk_* is the Pearson correlation coefficient between indicators *j* and *k*. The information content (*C_j_*) and the corresponding objective weight (*W_j_*) were calculated as:

C_j_ = S_j_R_j_ (25)

(26) $$W_{j}=\frac{C_{j}}{\sum_{k=1}^{m}C_{j}}$$

where the sum of all weights equals 1. The comprehensive score for the i-th object (*Q_i_*) was calculated as:

(27) $$Q_{i}=\sum_{j=1}^{m}W_{j}z_{\mathrm{ij}}$$

A higher *Q_i_* value represents superior comprehensive performance of the nutritional trajectory.

Primary scores were first calculated within each dimension, followed by a secondary CRITIC weighting of the eight-dimensional scores to obtain final rankings. Analysis was performed using custom MATLAB R2020a scripts and cross-verified with the MCDM Online platform to ensure precision to six decimal places.

### 2.12. Final Weight Prediction Model and Machine Learning Analysis

#### 2.12.1. Input Variables and Data Preprocessing

To analyze the non-linear relationship between initial body weight (BW), nutritional levels across three phases, and final BW, regression models including Backpropagation Neural Network (BPNN) [27,28], Support Vector Regression (SVR), and Random Forest (RF) [29] were constructed. Diets A, B, C, and D were encoded as 100, 90, 80, and 70, respectively, representing an ordinal gradient of comprehensive nutritional concentration. Only individuals with complete records for all three phases were included. The dataset was split into a training set and an independent test set (3:1) using stratified random sampling (random seed: 2024). Input variables were normalized using Min–Max scaling:

(28) $$x^{\prime }=\frac{x-x_{\min}}{x_{\max}-x_{\min}}$$

Parameters (*x_max_*, *x_min_*) were derived solely from the training set to prevent data leakage.

#### 2.12.2. NRBO Optimization Algorithm

A Newton–Raphson-Based Optimizer (NRBO) was used to optimize the hyperparameters for BPNN and SVR. NRBO utilizes Newton–Raphson search rules and a trap-avoidance mechanism to prevent local optima. The population size was set to 30 with a maximum of 100 iterations. The fitness function was defined as the 5-fold cross-validation root mean square error (RMSE) on the training set.

#### 2.12.3. NRBO-BPNN Model

The BPNN utilized a 4–10–1 topology (4 input neurons, 10 hidden neurons, 1 output neuron). The hidden layer used a hyperbolic tangent sigmoid activation function, while the output layer was linear. Training was performed using the Levenberg–Marquardt algorithm with an early stopping criterion based on a 15% internal validation set. NRBO optimized the initial weights and biases within a range of −1 to 1.

#### 2.12.4. NRBO-SVR Model

The SVR model employed a Radial Basis Function (RBF) kernel:

(29) $$K(x_{i},x_{j})\;=\;\exp(-\gamma ∥x_{i}-\;{x_{j}∥}^{2})$$

NRBO optimized the penalty parameter C ∈ [2^−5^, 2^15^] and the kernel parameter γ ∈ [2^−15^, 2^3^] on a logarithmic scale. The insensitivity parameter ϵ was fixed at 0.1.

#### 2.12.5. GS-RF Model

Hyperparameters for the RF model, including the number of trees (100–500), variables sampled at each split (1–4), and minimum leaf size (1–10), were optimized via Grid Search (GS) with 5-fold cross-validation. The criterion for optimization was the minimization of the average cross-validation RMSE.

#### 2.12.6. Model Performance Evaluation

Performance was evaluated using the coefficient of determination (R^2^) and the root mean square error (RMSE):

(30) $$R^{2}=1-\frac{\sum_{i=1}^{N}{\left(y_{i}-{\hat{y}}_{i}\right)}^{2}}{\sum_{i=1}^{N}(y_{i}-\bar{y}{)}^{2}}$$

(31) $$\mathrm{RMSE}=\sqrt{\frac{1}{N}\sum_{i=1}^{N}(y_{i}-{\hat{y}}_{i}}{)}^{2}$$

To assess stability, 100 repeated stratified random splits were performed to report the mean and 95% confidence interval of the performance metrics.

#### 2.12.7. SHAP Interpretation

Shapley Additive Explanations (SHAP) analysis was performed on the best-performing model (lowest test RMSE). Global importance was quantified using the mean absolute SHAP value:

(32) $$I_{j}=\frac{1}{N}\sum_{i=1}^{N}|\phi_{\mathrm{ij}}|$$

where *ϕ_ij_* is the SHAP value for variable j in sample i. TreeSHAP was used for RF, while KernelSHAP (with k-means clustering for representative background data) was used for SVR or BPNN. SHAP values were interpreted in kg after de-normalization to characterize the marginal effects of nutritional levels on final BW. 

### 2.13. Data Processing and Statistical Analysis

Raw data were sorted and organized using Microsoft Excel 2021. SAS 9.4 was applied for statistical analysis. The MCDM Online multi-criteria decision analysis platform was employed to implement the CRITIC approach, which was used to determine weights of primary and secondary indicators, compute comprehensive scores, and generate sample rankings. Machine learning analysis and figure visualization were carried out in MATLAB R2020a. Given that all pigs within a single pen received the same diet, the ‘pen’ was initially evaluated as a random effect in the preliminary mixed-model construction. However, because all pens were identical and located within a highly uniform, climate-controlled environment, the variance component associated with the pen effect was negligible. Therefore, to optimize model parsimony, the pen random effect was excluded from the final analysis, and the individual pig, based on the precise independent records from the single-space FIRE system, was utilized as the experimental unit. Distribution normality was assessed via Shapiro–Wilk tests and Q–Q plots. Non-normal data were transformed using natural logarithms or Box–Cox transformations. Missing data was not subjected to mean imputation. Statistical analysis of growth performance and physiological traits was performed using a general linear model (ANOVA), with dietary treatment as the fixed effect. All experimental data were verified for completeness, and no missing values were present in the final dataset. All analyses reported the actual sample size included and the explicit reasons for any exclusions. To ensure reproducibility, all machine learning models were fixed with a random seed of 2024. The sample indices for the training and test sets, normalization parameters, model architectures, optimal hyperparameters, and prediction results were rigorously documented and preserved. All data collected were verified for completeness, and there were no missing values in the dataset used for analysis.

## 3. Results

### 3.1. Summary of Key Growth Performance, Carcass, and Bone Traits

The primary biological responses of pigs to the dietary nutrient density treatments are summarized in Table 5. These foundational data served as the input for the subsequent CRITIC modeling and comprehensive evaluation. Detailed results for all 60+ measured indicators across the three growth phases, including means, SEM, and *p*-values, are provided in Supplementary Tables S1–S7.

### 3.2. Comprehensive Evaluation of Phase-Feeding Dietary Nutrient Concentrations Based on Growth Performance

Among the growth performance indicators, the weights assigned to final body weight and the feed conversion ratio were 0.424 and 0.576, respectively (Table 6 and Table S1). Although the weight of final body weight was slightly lower than that of the feed conversion ratio, the narrow margin between the two indicates that both metrics play a crucial role in the growth performance evaluation system.

The formula for calculating the comprehensive score of growth performance is as follows:

*Z* = 0.424*x*_1_ + 0.576*x*_2_

where x_1_ and x_2_ represent the standardized values for final body weight and the feed conversion ratio, respectively. Regarding growth performance, the comprehensive scores exhibited a wide range and pronounced differences among the treatment groups (Table 7). Specifically, the AAA and ABB groups achieved higher comprehensive scores of 0.885 and 0.758, respectively, demonstrating superior growth performance. Conversely, the DDA (0.149) and CCA (0.149) groups obtained lower scores, indicating relatively weaker growth performance.

### 3.3. Comprehensive Evaluation of Phase-Feeding Dietary Nutrient Concentrations Based on Nutrient Digestibility

Among the nutrient digestibility indicators, fat digestibility exhibited the highest weight (0.414), serving as the primary metric reflecting nutrient digestion, followed by protein digestibility (0.233). The weights assigned to fiber digestibility and energy digestibility showed minimal difference, ranking third and fourth, respectively (Table 8 and Table S2).

The comprehensive score for nutrient digestibility was calculated using the following formula:

*Z* = 0.189*x*_3_ + 0.414*x*_4_ + 0.233*x*_5_ + 0.165*x*_6_

where x_3_–x_6_ represent the standardized values for the digestibility of fiber, fat, protein, and energy, respectively. The DAA group achieved the highest score (0.925), indicating optimal nutrient digestibility, followed by the ABB (0.881) and ADA (0.834) groups, which also demonstrated robust nutrient utilization capabilities. Conversely, the CCC (0.322) and AAC (0.351) groups exhibited the lowest scores, reflecting the poorest nutrient utilization efficiency (Table 9).

### 3.4. Comprehensive Evaluation of Phase-Feeding Nutrient Concentration Regulation Based on Carcass Quality

Among the carcass-quality indicators, loin eye area received the highest weight (0.243), indicating that it was the most influential factor in the comprehensive carcass-quality evaluation. Backfat thickness ranked second, with a weight of 0.200. Dressing percentage (0.155), carcass straight length (0.140), skin thickness (0.135), and carcass diagonal length (0.126) had relatively similar weights, suggesting broadly comparable contributions to the carcass-quality evaluation (Table 10 and Table S3).

The composite score was calculated as follows:

*Z* = 0.155*x*_7_ + 0.140*x*_8_ + 0.126*x*_9_ + 0.243*x*_10_ + 0.200*x*_11_ + 0.135*x*_12_

where x_7_–x_12_ represent the standardized values for dressing percentage, carcass straight length, carcass diagonal length, loin eye area, backfat thickness, and skin thickness, respectively. The AAA trajectory achieved the highest composite score (0.654), indicating the most favorable overall carcass-quality performance. BBB (0.602) and ABB (0.587) ranked second and third, respectively, also demonstrating relatively strong overall performance. In contrast, CCA (0.403) and DDA (0.225) received the lowest scores, suggesting no clear advantage in carcass quality for these nutritional trajectories (Table 11).

### 3.5. Comprehensive Evaluation of Phase-Feeding Dietary Nutrient Concentrations Based on Bone Parameters

Based on the weight results of the bone parameters (Table 12 and Table S4), the weights of individual indicators ranged from 0.088 to 0.282, demonstrating pronounced overall differences. BS/BV exhibited the highest weight (0.282), serving as the pivotal indicator reflecting variations in bone parameters. BV/TV (0.205) and BS/TV (0.159) ranked second and third, respectively, suggesting that the relative proportion of bone tissue and the morphological characteristics of bone structure exerted significant impacts on the comprehensive evaluation. The weights of TV (0.136) and BV (0.130) were intermediate, whereas BS obtained the lowest weight (0.088), thereby contributing relatively less to the comprehensive evaluation.

The comprehensive score was calculated using the following formula:

*Z* = 0.130*x*_13_ + 0.136*x*_14_ + 0.205*x*_15_ + 0.088*x*_16_ + 0.159*x*_17_ + 0.282*x*_18_

where x_13_–x_18_ represent the standardized values for bone volume, tissue volume, bone volume fraction, bone surface area, bone surface to tissue volume ratio, and bone surface to bone volume ratio, respectively.

According to the comprehensive score results (Table 13), distinct nutritional trajectories exhibited significant differences in bone parameters. The ABB regimen achieved the highest comprehensive score (0.574), demonstrating the optimal overall performance in bone parameters under this nutritional trajectory. The DAA (0.535) and CCA (0.531) groups ranked second and third, respectively, showing robust performance. Conversely, the ACC (0.306) and DDA (0.310) groups ranked at the bottom, with ACC scoring the lowest, indicating a lack of apparent overall advantage in bone structural parameters.

### 3.6. Comprehensive Evaluation of Phase-Feeding Dietary Nutrient Concentrations Based on Trabecular Parameters

Based on the weight results of the trabecular-related indicators (Table 14 and Table S5), Tb. Th exhibited the highest weight (0.272), serving as the key factor influencing the comprehensive evaluation of the trabecular structure. Tb. N (0.236) and Tb. Sp (0.213) ranked second and third, respectively, exerting a significant impact on the comprehensive evaluation. The weights of SMI (0.143) and Tb. Pf (0.135) were relatively lower, yet they still contributed to the comprehensive assessment.

Z = 0.272x_19_ + 0.213x_20_ + 0.236x_21_ + 0.135x_22_ + 0.143x_23_

where x_19_–x_23_ represent the standardized values for trabecular thickness, trabecular separation, trabecular number, trabecular pattern factor, and structure model index, respectively. According to the results, the CCA regimen achieved the highest score (0.734), indicating the optimal overall performance in trabecular architecture under this nutritional trajectory. The ADA (0.689) and DDA (0.675) groups ranked second and third, respectively, demonstrating robust performance. Conversely, the ABB (0.520) and CCC (0.493) groups obtained relatively low scores, with the ACC group (0.391) ranking at the bottom and exhibiting the poorest performance (Table 15).

### 3.7. Comprehensive Evaluation of Phase-Feeding Dietary Nutrient Concentrations Based on Bone Density

Based on the weight results of the bone density-related indicators (Table 16 and Table S6), the weight distribution among the indicators was relatively balanced, ranging from 0.225 to 0.274. TMD exhibited the highest weight (0.274), serving as the core indicator influencing the comprehensive evaluation of bone density; BMD (0.266) ranked second, indicating that bone mineral density is equally of significant importance to the evaluation. TMC (0.234) and BMC (0.225) ranked third and fourth, respectively, with a marginal difference in their weights.

*Z* = 0.266*x*_24_ + 0.225*x*_25_ + 0.274*x*_26_ + 0.234*x*_27_

where x_24_–x_27_ represent the standardized values for bone mineral density, bone mineral content, tissue mineral density, and tissue mineral content, respectively.

According to the comprehensive scores of the different nutritional trajectories (Table 17), the CCA regimen achieved the highest score (0.729), indicating optimal overall performance in bone density under this nutritional trajectory. The ADA (0.625) and BBB (0.555) groups ranked second and third, respectively, exhibiting relatively higher bone density levels. Conversely, the CCC (0.395), DDA (0.404), and ACC (0.408) groups obtained lower scores, with the AAC group (0.243) ranking at the bottom, suggesting a lack of apparent overall advantage in bone density-related parameters for these regimens.

### 3.8. Comprehensive Evaluation of Phase-Feeding Dietary Nutrient Concentrations Based on Body Measurements

Among the body measurement indicators, the weight distribution was relatively balanced, ranging from 0.169 to 0.240 (Table 18 and Table S7). Specifically, RH exhibited the highest weight (0.240), making the greatest contribution to differentiating body measurement variations among the different nutritional trajectories. CH (0.208) and RW (0.193) ranked second and third, respectively, indicating that both longitudinal and transverse developmental indicators exerted significant impacts on the comprehensive evaluation. CW (0.190) and BL (0.169) obtained relatively lower weights, suggesting a harmonious influence of the respective indicators on the evaluation outcomes.

*Z* = 0.169*x*_28_ + 0.240*x*_29_ + 0.193*x*_30_ + 0.208*x*_31_ + 0.190*x*_32_

where x_28_–x_32_ represent the standardized values for body length, rump height, rump width, chest height, and chest width, respectively.

Pronounced differences were observed in the comprehensive scores of body measurements among the distinct nutritional trajectories (Table 19). The AAA regimen achieved the highest score (0.578), ranking first and demonstrating optimal overall performance in body dimension development. The ABB (0.525) and AAB (0.516) groups ranked second and third, respectively, exhibiting apparent advantages in body measurements. The AAC (0.508) and BBB (0.498) groups remained at an intermediate level. Conversely, the DDA group recorded the lowest comprehensive score (0.102), indicating relatively weaker body dimension development.

### 3.9. Comprehensive Evaluation of Phase-Feeding Dietary Nutrient Density Trajectories Based on Energy and Nitrogen Deposition

The weights of the secondary indicators for energy and nitrogen deposition were relatively balanced (Table 20 and Table S8). The apparent nitrogen digestibility was assigned the highest weight (0.403), representing the most significant contributor to differentiating the variations in energy and nitrogen deposition. Energy deposition rate (0.339) and nitrogen deposition rate (0.258) ranked second and third, respectively.

The comprehensive index was calculated using the following formula:

*Z* = 0.339*x*_33_ + 0.403*x*_34_ + 0.258*x*_35_

Among these, x_33_, x_34_ and x_35_ represent the standardized values for energy deposition rate, apparent nitrogen digestibility, and nitrogen deposition rate, respectively. The ABB trajectory achieved the highest score (0.854), indicating optimal deposition efficiency. The scores for BBB (0.766) and DAA (0.764) were marginally different, ranking second and third, respectively. ADA (0.706), DDA (0.702), and CCA (0.666) all ranked among the top, demonstrating significant compensatory effects. In contrast, ACC (0.419), CCC (0.409), and AAC (0.407) performed poorly, highlighting the pronounced adverse effects of these specific nutritional regulation strategies (Table 21).

### 3.10. Weights and Evaluation of Primary Indicators

**Figure 3:**
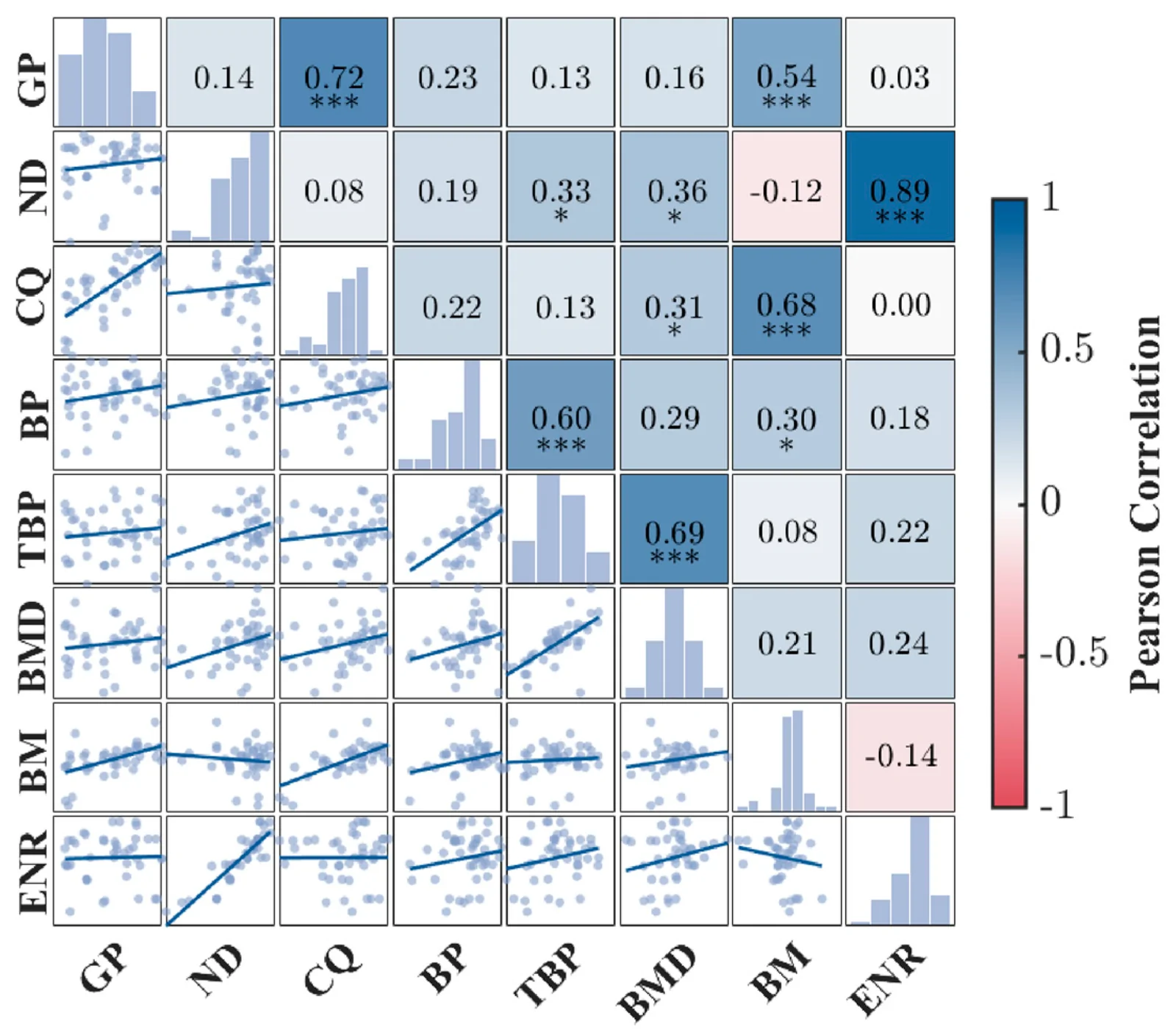
Heatmap of Pearson correlations among primary indicators. Note: For the Pearson correlation analysis, * indicates *p* < 0.05 and *** indicates *p* < 0.001.

The Pearson correlations among the primary indicators are illustrated in Figure 3. As depicted, the correlation coefficient between nutrient digestibility and energy and nitrogen retention was 0.89 (*p* < 0.001). The correlation coefficients of growth performance with carcass quality and body measurements were 0.72 and 0.54, respectively (*p* < 0.001). The correlation coefficient between carcass quality and body measurements was 0.68 (*p* < 0.001). Furthermore, the correlation coefficient between trabecular parameters and bone density was 0.69 (*p* < 0.001), while that between overall bone parameters and trabecular parameters was 0.60 (*p* < 0.001). The correlation coefficients of nutrient digestibility with trabecular parameters and bone density were 0.33 and 0.36, respectively (*p* < 0.05). The correlation coefficient between carcass quality and bone density was 0.31 (*p* < 0.05), and the correlation between bone parameters and body measurements was 0.30 (*p* < 0.05). Correlations among the remaining indicators were relatively weak, with some failing to reach statistical significance.

Scatter plots and fitted regression lines further demonstrated a linear relationship among most traits, although the strength of these correlations varied. This suggests that while the indicators exhibit a trend of synergistic variation, they also retain a certain degree of independence.

As shown in Table 22, the weights of the primary indicators ranged from 0.098 to 0.162, indicating a relatively balanced overall distribution. Specifically, the growth performance indicator exhibited the highest weight (0.162), indicating that growth performance made the greatest contribution to the comprehensive evaluation system. The weights for energy and nitrogen retention (0.150) and carcass quality (0.126) ranked second and third, respectively, demonstrating their significant impact on the overall evaluation. The weight for trabecular parameters (0.124) ranked fourth. The weights for bone parameters (0.118), nutrient digestibility (0.117), body measurements (0.104), and bone density (0.098) were relatively lower. The minimal margin of difference among these weights suggests that all indicators exerted a relatively stable role within the evaluation system. Herein, all parameters represent standardized data.

According to the comprehensive scores of the different nutritional trajectories (Table 23), the ABB regimen achieved the highest score (0.717), demonstrating optimal overall performance. The AAA (0.690) and BBB (0.643) groups ranked second and third, respectively, exhibiting a high overall performance level. Conversely, the AAC (0.445), ACC (0.387), and CCC (0.386) obtained lower scores, with the DDA group (0.359) ranking at the bottom. Pronounced differences were observed in the comprehensive performance of multi-dimensional indicators across the distinct nutritional trajectories, among which the ABB regimen demonstrated the most prominent advantage.

### 3.11. Effects of Phase-Feeding Dietary Nutrient Concentrations on Final Body Weight of Growing–Finishing Pigs

**Figure 4:**
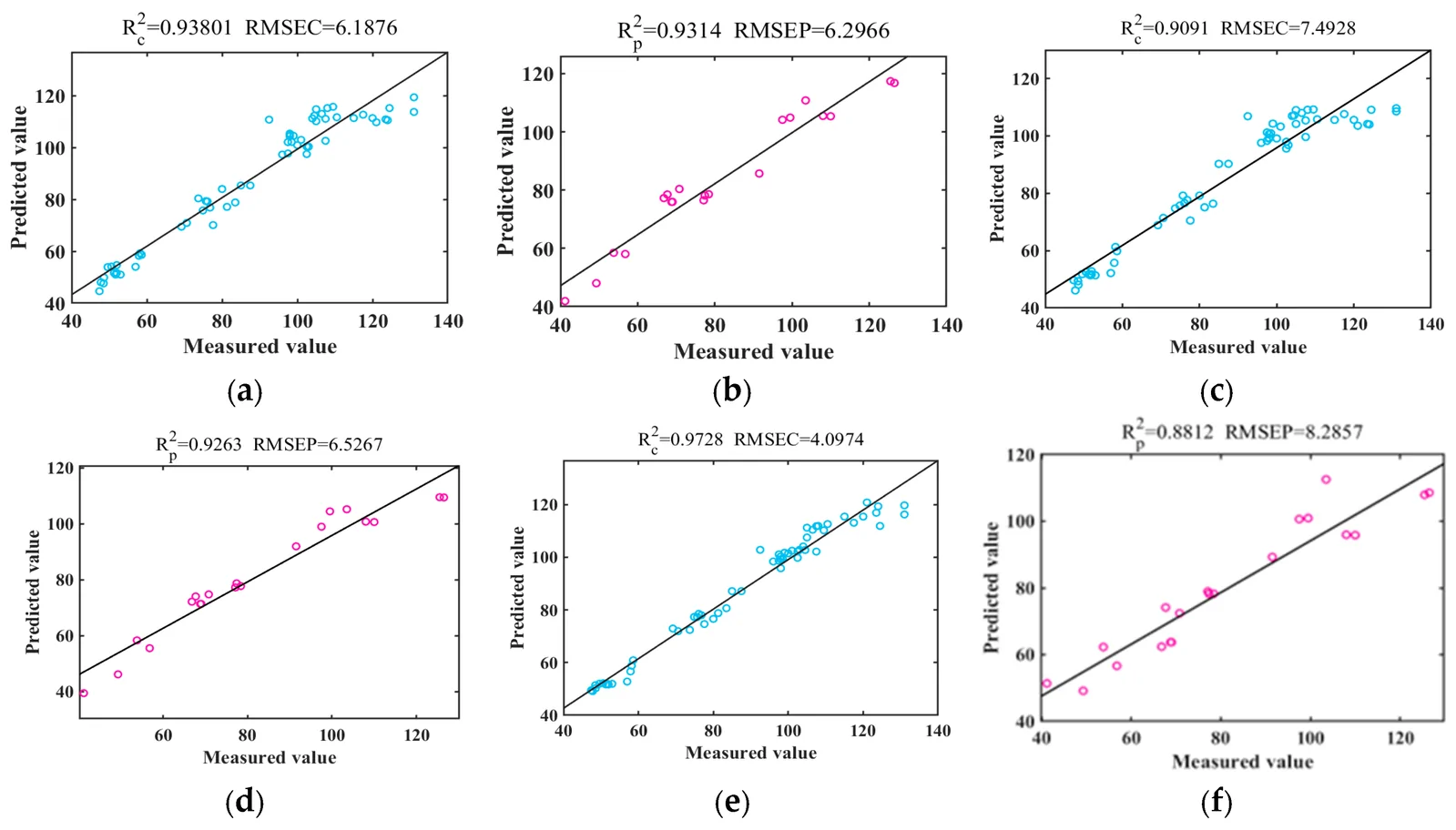
Modeling results of three models: (**a**) NRBO-BPNN training results; (**b**) NRBO-BPNN testing results; (**c**) NRBO-SVR training results; (**d**) NRBO-SVR testing results; (**e**) GS-RF training results; (**f**) GS-RF testing results.

The NRBO-SVR, NRBO-BPNN, and GS-RF models effectively predicted the final body weight (Figure 4). The R^2^ values of the training sets for all three models were above 0.90, and the R^2^ values of the test sets were above 0.88. Among them, the NRBO-BPNN model exhibited the best performance, yielding a training set Rc2 and RMSE of 0.9380 and 6.1876 kg, respectively (Figure 4a), and a test set Rp2 and RMSE of 0.9314 and 6.2966 kg, respectively (Figure 4b).

**Figure 5:**
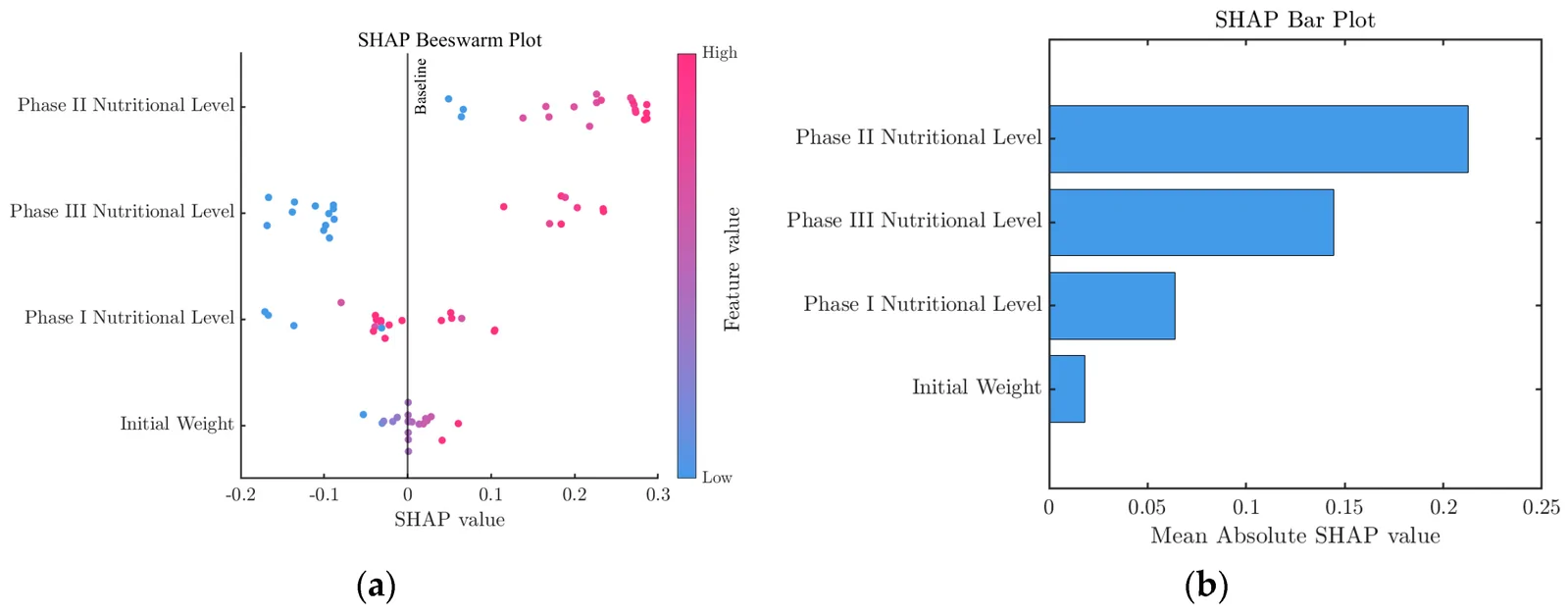
SHAP analysis plots: (**a**) summary plot; (**b**) bar plot.

As illustrated in the SHAP summary plot (Figure 5a), there were pronounced differences in the direction and magnitude of the impact of various predictors on the model output. Specifically, the Phase 2 nutritional level was predominantly clustered in the positive region, indicating that higher values exerted a more pronounced promoting effect on the predicted results, making it the primary positive driver. The Phase 3 nutritional level also demonstrated a positive contribution; however, the impact of the Phase 1 nutritional level was relatively weak, suggesting it had a minor influence on the model predictions.

The SHAP bar chart (Figure 5b) further quantified the ranking of variable importance based on the mean absolute SHAP values. The results revealed the following sequence: Phase 2 nutritional level > Phase 3 nutritional level > Phase 1 nutritional level > initial body weight. This indicates that the model was primarily driven by the nutritional levels during the middle and late stages, whereas the initial body weight provided a limited contribution to the overall prediction.

## 4. Discussion

Skeletal, muscular, and adipose tissues exhibit distinct developmental timelines and nutrient requirements during the growing–finishing period, and their physiological responses to phase-restricted nutrient intake and subsequent nutritional supplementation are asynchronous [30,31,32]. The results of the present study demonstrate that the biological effects of dynamic nutritional trajectories are markedly trait-specific and phase-dependent [33]. Certain trajectories achieved superior growth performance and morphometric body measurements but showed no competitive advantage in skeletal microarchitecture or energy and nitrogen retention, indicating that FBW or average daily gain (ADG) failed to fully reflect the comprehensive developmental status of individual tissues and organs [34].

The ADA trajectory (100% → 70% → 100%) yielded superior outcomes in several trabecular bone microarchitectural indices and BMD-related parameters without compromising morphometric body measurements or growth performance. This suggests that nutritional re-alimentation following mid-phase restriction favors skeletal microarchitecture and mineralization while maintaining subsequent growth performance. Nutritional re-alimentation following phase-restricted feeding can trigger compensatory adaptations in bone formation and mineralization; however, the magnitude of such compensation is co-regulated by the severity, duration, and timing of restriction, as well as the dietary nutrient profile [35]. The skeletal responses observed in the ADA trajectory align with this potential compensatory mechanism. Because bone histology and molecular biomarkers associated with osteogenesis and osteoclast genesis were not assayed, the precise cellular and physiological mechanisms remain to be determined.

The DDA trajectory (70% → 70% → 100%) exhibited enhanced nutrient digestibility with superior energy and nitrogen retention following late-phase nutritional re-alimentation, yet its terminal body measurements and growth performance were suppressed. This suggests that digestive and metabolic traits possess pronounced short-term plasticity, whereas morphometric growth and skeletal development are more vulnerable to early nutritional history and long-term cumulative tissue deposition. Persistent early-phase nutrient deficiency may exert enduring negative effects on skeletal framework development that cannot be entirely reversed by subsequent re-alimentation [36]. Given that growth plates, dynamic bone formation rates, and systemic endocrine regulatory axes were not directly quantified, the precise mechanisms through which early-phase nutrient restriction impairs skeletal framework ontogeny warrant further investigation.

Overall, the findings suggest that compensatory nutritional responses are both trait-specific and phase-dependent. Nutrient digestibility and energy/nitrogen retention respond relatively rapidly to late-phase re-alimentation. Trabecular microarchitecture is sensitive to mid-to-late nutrient provision, although the degree of recovery is contingent on the early-phase nutritional foundation; conversely, body measurements and overall growth are more susceptible to early phase and prolonged nutrient regimens. Therefore, relying exclusively on FBW or ADG to evaluate dynamic nutritional strategies may obscure disparities in skeletal framework integrity and nutrient utilization, highlighting the necessity of integrating both acute production performance and long-term structural phenotypes [37,38].

It is important to note that the use of corncob powder as an inert diluent inherently increased the dietary fiber content in the restricted diets. The elevated fiber levels likely contributed to the observed reductions in apparent total tract digestibility (ATTD) of nutrients during the restricted phases [39], as high dietary fiber accelerates intestinal transit time and can physically hinder the access of endogenous digestive enzymes to nutrients [40]. Furthermore, while energy and amino acids were diluted, the absolute concentrations of calcium and phosphorus were maintained across all diets. Consequently, the mineral-to-energy ratio was markedly higher in the restricted (diluted) diets. This altered ratio meant that pigs fed the restricted diets, despite having lower energy intakes, consumed relatively enough osteogenic minerals per unit of energy [41]. This can explain why the severe nutrient dilution (e.g., 70% trajectory) had a disproportionately milder detrimental effect on fundamental bone geometric dimensions compared to soft tissue (fat and muscle) deposition, as the preferential partitioning of available minerals toward skeletal mineralization was supported by the enriched mineral-to-energy ratio [42].

To reduce the bias inherent in any single-indicator evaluation, we constructed a multidimensional comprehensive evaluation framework based on the CRITIC (CRiteria Importance Through Inter-criteria Correlation) method [43,44]. The resulting weight-allocation analysis showed that growth performance and energy/nitrogen retention made the largest contributions, indicating that these indicators possess strong discriminative power in distinguishing among distinct nutritional trajectories. Carcass quality and trabecular microarchitectural parameters also carried substantial weight. These findings confirm that economic yield, nutrient-retention efficiency, and skeletal integrity are indispensable dimensions for assessing phase-feeding strategies [45]. Phase feeding—feeding several diets in sequence, each formulated to match the pig’s nutrient requirements at the average body weight within a given phase—aims to shorten the periods during which pigs are supplied with either deficient or excessive nutrients. Notably, microstructural metrics such as trabecular thickness can capture granular biological variation that gross body weight, body size, and bone mass fail to reveal, thereby increasing the analytical resolution of skeletal structural evaluation [46].

In this study, the CRITIC weight analysis assigned the second-highest weight to the ‘Energy and Nitrogen Deposition’ domain, despite its high correlation (r = 0.89) with the ‘Nutrient Digestibility’ domain. According to the CRITIC weighting logic, while high inter-criteria correlation tends to penalize the weight due to redundancy, the final weight is also a function of the contrast intensity (standard deviation) of the data. The high weight assigned to energy/N deposition reflects its high sensitivity and information volume in response to dietary treatments. Although apparent N digestibility and protein digestibility describe similar processes, the former—when integrated into the energy/N deposition domain—incorporates metabolic utilization and urinary losses, providing a more sensitive indicator of the physiological response to varying nutrient densities in the modeling process.

Accurate prediction of FBW is instrumental in guiding precise phase-feeding management for growing–finishing pigs. In this study, we benchmarked three predictive algorithms: NRBO-BPNN, NRBO-SVR, and GS-RF. NRBO-BPNN demonstrated robust performance consistency across the training and test sets, achieving a favorable balance between fitting accuracy and generalization capability. The predictive performance of NRBO-SVR was slightly lower than that of NRBO-BPNN, although it maintained overall stability. Conversely, GS-RF displayed noticeable discrepancies between the training and test sets and suffered substantial prediction deviations in higher actual FBW domains, suggesting potential model overfitting or inadequate generalization of high-weight samples within this dataset. Accordingly, under the specific breed, weight window, housing conditions, and dietary treatments in this trial, NRBO-BPNN emerged as the preferred model for FBW prediction. However, its cross-cohort and cross-scenario transferability requires independent, multi-batch validation.

SHAP feature attribution analysis indicated that dietary nutrient levels in Phases 2 and 3 exerted higher predictive contributions to FBW than Phase 1 nutrient density or initial BW [47]. The SHAP values of the Phase 2 nutrient variables were predominantly positive with a broad distribution, indicating that higher Phase 2 nutrient density was strongly correlated with elevated predicted FBW and accounted for substantial inter-individual predictive variation. The Phase 3 nutrient variable showed a consistently positive impact with a more concentrated SHAP distribution, reflecting its stable contribution to the terminal weight prediction. The Phase 1 nutrient variables exhibited alternating positive and negative SHAP values of lower magnitude, and the initial BW clustered narrowly near zero, suggesting marginal predictive leverage in the final model.

SHAP values quantify the conditional contributions to model outputs and do not inherently denote biological or physiological causality. The limited variance in initial BW, inter-collinearity among phase nutrient variables, and categorical/numerical encoding of trajectories could all bias the feature importance attribution. Therefore, these findings should not be interpreted as indicating that the initial BW or Phase 1 nutrition lacks biological significance for subsequent swine life stages. The prominent predictive contribution of Phase 2 nutrition likely stems from its timing at the critical transition between rapid frame elongation and active fattening; Phase 3 nutrition directly drives tissue accretion immediately prior to slaughter and exerts an immediate and stable effect on FBW. Although Phase 1 nutrition dictates foundational skeletal and visceral organogenesis, its direct mathematical contribution to terminal BW is likely masked or diluted by subsequent reorientation and compensatory growth dynamics.

Synthesizing machine-learning and CRITIC comprehensive evaluation results shows that mid-to-late nutrient provision is a pivotal determinant of terminal weight and overall production performance under the current experimental conditions. Predictive modeling and multimeric comprehensive evaluation address fundamentally distinct scientific questions. The former forecasts terminal weight per se, whereas the latter seeks equilibrium across growth, carcass quality, skeletal integrity, and metabolic efficiency. Therefore, the predictive value of FBW cannot be conflated with superior skeletal robustness or nutrient retention.

Treatments A, B, C, and D in this study represented multi-nutrient package dilutions. Because these formulations concurrently modified metabolizable energy, CP, and amino acid profiles, SHAP analysis captured the integrated predictive contribution of phase-specific macronutrient packages rather than the isolated effect of an individual amino acid. Integrating terminal weight, feed cost dynamics, carcass yield, skeletal microarchitecture, nitrogen emission footprints, and animal welfare metrics into a unified multi-objective optimization framework is vital for comprehensively advancing precise dynamic protein and amino acid feeding strategies.

## 5. Conclusions

Our findings elucidate the critical, time-dependent impacts of nutrient density on skeletal and metabolic ontogeny, offering stage-specific strategies for precision production. Persistence of Early- and Late-Phase Deprivation: Early-phase nutrition strongly dictates the skeletal structural foundation, where severe restriction causes morphometric and growth deficits that are not fully compensated by the time pigs reach market weight. Metabolic Plasticity vs. Growth Continuity: Nutrient digestibility and metabolic retention exhibit high compensatory plasticity upon late-phase re-alimentation, even following severe early deprivation. In contrast, maximizing absolute growth performance demands uninterrupted nutritional continuity throughout the entire developmental trajectory. Objective-Driven Nutritional Strategies: To maximize lean carcass yield and absolute body weight, uninterrupted high-density nutrition or very mild mid-phase restriction is optimal. Conversely, to prioritize skeletal integrity and bone frame development (e.g., for producing robust finishing pigs), a compensatory regimen—moderate early-to-mid restriction paired with late-phase reinforcement—proves more effective. Predictive Drivers of Terminal Growth: Multidimensional evaluations reveal that mid-phase nutrient density is the primary driver of terminal slaughter weight. The most balanced composite outcome was achieved through a temporally optimized trajectory involving high early-phase provision paired with moderate late-phase dilution (e.g., the ABB trajectory). Although this strategy still involves nutrient dilution in the later phases, it provides the optimal trade-off between growth efficiency, nutrient utilization, and skeletal health under our evaluation framework.

## Figures and Tables

**Table 1:** Composition and nutrient levels of diets.

Items	25–50 kg				50–75 kg				75–100 kg		
	100%	90%	80%	70%	100%	90%	80%	70%	100%	90%	80%
Corn (g)	727.90	655.11	582.32	509.53	742.40	668.16	593.92	519.68	673.90	606.51	539.12
Soybean meal (g)	210.00	189.00	168.00	147.00	170.00	153.00	136.00	119.00	110.00	99.00	88.00
Wheat middlings (g)	—	—	—	—	—	—	—	—	120.00	108.00	96.00
Wheat bran (g)	20.00	18.00	16.00	14.00	50.00	45.00	40.00	35.00	60.00	54.00	48.00
Sodium chloride (g)	5.00	5.00	5.00	5.00	5.00	5.00	5.00	5.00	5.00	5.00	5.00
Limestone (g)	12.00	12.40	12.90	13.40	12.00	12.30	12.70	13.00	12.00	12.30	12.60
Dicalcium phosphate (g)	9.00	9.10	9.30	9.40	5.00	5.10	5.20	5.30	3.70	3.80	3.90
L-lysine hydrochloride (g)	3.30	2.97	2.64	2.31	3.40	3.06	2.72	2.38	3.50	3.15	2.80
DL-methionine (g)	1.20	1.08	0.96	0.84	0.90	0.81	0.72	0.63	0.70	0.63	0.56
L-threonine (g)	1.20	1.08	0.96	0.84	1.20	1.08	0.96	0.84	1.10	0.99	0.88
L-tryptophan (g)	0.20	0.18	0.16	0.14	0.10	0.09	0.08	0.07	0.10	0.09	0.08
L-valine (g)	0.20	0.18	0.16	0.14	—	—	—	—	—	—	—
1% premix (g)	10.00	10.00	10.00	10.00	10.00	10.00	10.00	10.00	10.00	10.00	10.00
Corn cob (g)	—	95.90	191.60	287.40	—	96.40	192.70	289.10	—	96.53	193.06
Total (g)	1000.00	1000.00	1000.00	1000.00	1000.00	1000.00	1000.00	1000.00	1000.00	1000.00	1000.00
Nutrient levels											
ME (kcal/kg)	3165.82	2989.06	2812	2635.08	3165.07	2989.1	2813.01	2637.04	3115.89	2945.03	2774.18
Moisture (%)	12.47	11.85	11.23	10.63	12.57	11.95	11.33	10.71	12.74	12.1	11.46
Crude Protein (%)	15.45	14.13	12.8	11.48	14.26	13.05	11.85	10.64	13.35	12.23	11.12
NDF (%)	10.56	15.63	20.69	25.75	11.36	16.38	21.4	26.42	12.5	17.42	22.34
Ash (%)	5.51	5.18	4.85	4.52	5.14	4.85	4.56	4.26	5.11	4.82	4.53
Fat (%)	2.9	2.7	2.49	2.29	2.99	2.78	2.56	2.35	3.18	2.95	2.72
ADF (%)	3.68	6.85	10.02	13.19	3.85	7.03	10.2	13.36	4.24	7.38	10.51
Calcium (%)	0.74	0.74	0.74	0.74	0.64	0.64	0.64	0.64	0.61	0.61	0.61
Phosphorus (%)	0.48	0.48	0.48	0.48	0.42	0.42	0.42	0.42	0.45	0.45	0.45
SID Lys (%)	1.01	0.91	0.80	0.70	0.89	0.80	0.71	0.63	0.80	0.72	0.64
Digestible Phos (%)	0.29	0.29	0.29	0.29	0.26	0.26	0.26	0.26	0.25	0.25	0.25
SID M + C (%)	0.56	0.50	0.45	0.39	0.50	0.45	0.40	0.35	0.47	0.42	0.37
SID Met (%)	0.34	0.30	0.27	0.24	0.30	0.27	0.24	0.21	0.27	0.24	0.21
SID Thr (%)	0.60	0.54	0.48	0.42	0.56	0.50	0.45	0.39	0.51	0.46	0.40
SID Trp (%)	0.18	0.16	0.14	0.13	0.15	0.14	0.12	0.11	0.14	0.12	0.11
SID Val (%)	0.66	0.59	0.53	0.46	0.59	0.53	0.47	0.41	0.54	0.48	0.43

Note: Each kilogram of the premix provides the following vitamins and minerals: Vitamin A 28,500 IU; Vitamin D 36,000 IU; Vitamin E 67.5 IU; Vitamin K 37.5 mg; Vitamin B1 17.5 mg; Vitamin B2 215 mg; Vitamin B6 69 mg; Vitamin B12 0.075 mg; Niacin 25 mg; Folic acid 3 mg; Calcium pantothenate 0.375 mg; Antioxidant 0.15 mg; Choline chloride 105 mg; Cobalt sulfate anhydrous 1 mg; Copper sulfate pentahydrate 155 mg; Ferrous sulfate 145 mg; Manganese monoxide 75 mg; Zinc sulfate 125 mg; Potassium iodide 0.3 mg; Sodium selenite 0.3 mg.

**Table 2:** The nutrients content of experimental feedstuff.

Items	Corn Cob	Corn	Soybean Meal	Wheat Middlings	Wheat Bran
Crude fiber (%)	37.1	1.9	2.3	3.5	4.1
Ether extract (%)	0.9	4.9	1	3.8	3.8
Crude protein (%)	2.3	6.5	40.6	14.6	13.6
Moisture (%)	6.6	12	7.3	10.8	7.1
Ash (%)	2.3	1.2	6.3	3.5	4.2
Nitrogen-free extract (%)	50.8	73.5	42.5	63.8	67.2
Cellulose (%)	30.6	3.7	2.3	7.6	9
Hemicellulose (%)	27.0	6.5	4.3	23.8	37.1
Lignin (%)	6.3	0.6	0.18	1.7	4.0
Standardized ileal digestibility (%)	18.0	88.0	84.0	92.0	72.0
Phosphorus digestibility (%)	12.1	34.0	47.0	52.3	54.2
Gross energy (MJ/kg)	17.2	16.2	17.4	16.1	17.1
Metabolizable energy (MJ/kg)	6.1	14.3	15.0	13.7	11.0

**Table 3:** Micro-CT scanning parameters.

Items	Value	Unit
Tube voltage	80	kV
Tube current	0.06	mA
Source-to-detector distance (SDD)	410	mm
Source-to-sample distance (SSD)	90	mm
Frames per second	20	frames/s
Total frames	4000	frames
Transverse FOV	51	mm
Axial FOV	16.014	mm
Voxel size	51	μm
CT threshold	783.3	—

**Table 4:** Evaluation indicators and indicator directions.

Primary Indicator	Secondary Indicator	Unit	Indicator Direction	Primary Indicator	Secondary Indicator	Unit	Indicator Direction
Growth performance	Final weight	kg	+	Trabecular bone	Trabecular thickness (Tb. Th)	mm	+
	Feed-to-gain ratio (F/G)		−		Trabecular separation (Tb. Sp)	mm	−
Nutrient digestibility	Fiber digestibility	%	+		Trabecular number (Tb. N)	mm	+
	Fat digestibility	%	+		Trabecular pattern factor (Tb. Pf)	mm	−
	Protein digestibility	%	+		Structure model index (SMI)		−
	Energy digestibility	%	+	Bone density	Bone mineral density (BMD)	mg/cm^3^	+
Carcass traits	Dressing percentage	%	+		Bone mineral content (BMC)	mg	+
	Carcass straight length	cm	+		Tissue mineral density (TMD)	mg/cm^3^	+
	Carcass diagonal length	cm	+		Tissue mineral content (TMC)	mg	+
	Loin eye area	cm^2^	+	Body measurement	Body length	cm	+
	Backfat thickness	cm	+		Rump height	cm	+
	Skin thickness	cm	−		Rump width	cm	+
Bone parameters	Bone volume (BV)	mm^3^	+		Chest height	cm	+
	Tissue volume (TV)	mm^3^	+		Chest width	cm	+
	Bone volume fraction (BV/TV)		+	Energy and nitrogen deposition	Energy deposition rate	%	+
	Bone surface (BS)	mm^2^	+		Apparent nitrogen digestibility	%	+
	Bone surface to tissue volume ratio (BS/TV)	mm^−1^	+		Nitrogen deposition rate	%	+
	Bone surface to bone volume ratio (BS/BV)	mm^−1^	+				

**Table 5:** Key performance and physiological traits of pigs (75–100 kg phase).

Treatment	BMD	BMC	TMD	TMC	ADG	ADFI	FCR	Dressing Percentage	Skin Thickness	Backfat Thickness	Carcass Straight Length	Carcass Diagonal Length	Loin Eye Area
	mg/cm^3^	mg	mg/cm^3^	mg	kg	kg		%	cm	cm	cm	cm	cm^2^
AAA	195.4	366.0	402.8 ^ab^	293.2	0.965 ^a^	2.617 ^bcd^	2.736 ^c^	73.1 ^ab^	0.34 ^b^	1.88 ^a^	100.10 ^a^	112.20 ^ab^	35.77 ^abc^
BBB	172.6	324.5	373.4 ^abc^	251.1	0.958 ^a^	2.780 ^ab^	2.911 ^c^	71.5 ^abc^	0.34 ^b^	1.51 ^abc^	97.67 ^abc^	111.00 ^abc^	42.26 ^ab^
CCC	170.5	414.4	380.2 ^abc^	330.1	0.711 ^cd^	2.552 ^bcd^	3.595 ^a^	69.8 ^cd^	0.36 ^b^	1.23 ^bc^	94.00 ^bcd^	105.33 ^cde^	43.66 ^ab^
ABB	177.4	308.7	388.5 ^ab^	240.0	0.961 ^a^	2.990 ^ab^	3.112 ^abc^	73.5 ^a^	0.40 ^ab^	1.16 ^cd^	99.00 ^ab^	113.33 ^a^	36.35 ^abc^
ACC	166.9	329.0	362.2 ^bc^	262.6	0.686 ^cd^	2.172 ^d^	3.170 ^abc^	70.8 ^bcd^	0.33 ^b^	0.62 ^d^	93.67 ^cd^	105.00 ^cde^	48.71 ^a^
AAB	155.5	278.4	332.2 ^c^	226.7	0.720 ^cd^	2.153 ^d^	3.012 ^abc^	70.6 ^cd^	0.37 ^ab^	1.54 ^abc^	94.67 ^bcd^	106.33 ^bcd^	34.28 ^abc^
AAC	226.7	421.4	417.9 ^a^	343.4	0.630 ^d^	2.262 ^cd^	3.588 ^a^	68.5 ^d^	0.37 ^ab^	1.31 ^abc^	93.67 ^cd^	103.33 ^de^	42.21 ^ab^
CCA	193.4	364.8	379.8 ^abc^	287.2	0.793 ^bc^	2.814 ^ab^	3.558 ^ab^	70.4 ^cd^	0.34 ^b^	1.28 ^bc^	94.33 ^bcd^	105.33 ^cde^	28.35 ^bc^
DAA	186.8	284.2	391.6 ^ab^	226.7	0.926 ^ab^	2.692 ^abc^	2.914 ^bc^	71.5 ^abc^	0.36 ^b^	1.81 ^ab^	97.33 ^abc^	107.67 ^abcd^	36.26 ^abc^
DDA	213.2	387.2	405.4 ^ab^	307.6	0.996 ^a^	3.026 ^ab^	3.037 ^abc^	68.5 ^d^	0.46 ^a^	1.01 ^cd^	91.67 ^d^	99.67 ^e^	25.39 ^c^
ADA	4.497	10.532	4.547	8.856	1.002 ^a^	3.149 ^a^	3.133 ^abc^	71.9 ^abc^	0.35 ^b^	1.51 ^abc^	94.00 ^bcd^	105.67 ^cde^	40.05 ^abc^
SEM	0.084	0.084	0.015	0.097	0.023	0.061	0.066	0.273	0.007	0.055	0.646	0.806	1.593
*p*-value	BMD	BMC	TMD	TMC	0.000	0.000	0.008	0.000	0.025	0.000	0.000	0.000	0.035

Note: In the same row, values with different letter superscripts mean significant difference (*p* < 0.05), while with same letter or no letter superscripts mean no significant difference (*p* > 0.05).

**Table 6:** Weight of secondary indicators for growth performance.

Indicator	Weight	Rank
Feed conversion ratio	0.576	1
Final body weight	0.424	2

**Table 7:** Comprehensive score and ranking of growth performance in each treatment group.

Treatment	Comprehensive Score	Rank
AAA	0.885	1
ABB	0.758	2
AAB	0.643	3
ADA	0.613	4
BBB	0.540	5
AAC	0.471	6
ACC	0.313	7
DAA	0.312	8
CCC	0.155	9
CCA	0.149	10
DDA	0.149	10

**Table 8:** Weight of secondary indicators for nutrient digestibility.

Indicator	Weight	Rank
Fat digestibility	0.414	1
Protein digestibility	0.233	2
Fiber digestibility	0.189	3
Energy digestibility	0.165	4

**Table 9:** Comprehensive score and ranking of nutrient digestibility in each treatment group.

Treatment	Comprehensive Score	Rank
DAA	0.925	1
ABB	0.881	2
ADA	0.834	3
BBB	0.801	4
DDA	0.779	5
CCA	0.749	6
AAA	0.685	7
AAB	0.646	8
ACC	0.587	9
AAC	0.351	10
CCC	0.322	11

**Table 10:** Weight of secondary indicators for carcass quality.

Indicator	Weight	Rank
Loin eye area	0.243	1
Backfat thickness	0.200	2
Dressing percentage	0.155	3
Carcass straight length	0.140	4
Skin thickness	0.135	5
Carcass diagonal length	0.126	6

**Table 11:** Comprehensive score and ranking of carcass quality in each treatment group.

Treatment	Comprehensive Score	Rank
AAA	0.654	1
BBB	0.602	2
ABB	0.587	3
DAA	0.558	4
ADA	0.529	5
ACC	0.488	6
CCC	0.478	7
AAB	0.470	8
AAC	0.432	9
CCA	0.403	10
DDA	0.225	11

**Table 12:** Weights of secondary indicators for bone parameters.

Indicator	Weight	Rank
BS/BV	0.282	1
BV/TV	0.205	2
BS/TV	0.159	3
TV	0.136	4
BV	0.130	5
BS	0.088	6

**Table 13:** Comprehensive score and ranking of bone parameters in each treatment group.

Treatment	Comprehensive Score	Rank
ABB	0.574	1
DAA	0.535	2
CCA	0.531	3
AAB	0.527	4
AAC	0.513	5
AAA	0.499	6
CCC	0.487	7
ADA	0.481	8
BBB	0.414	9
DDA	0.310	10
ACC	0.306	11

**Table 14:** Weights of secondary indicators for bone trabeculae.

Indicator	Weight	Rank
Tb. Th	0.272	1
Tb. N	0.236	2
Tb. Sp	0.213	3
SMI	0.143	4
Tb. Pf	0.135	5

**Table 15:** Comprehensive score and ranking of trabecular bone in each treatment group.

Treatment	Comprehensive Score	Rank
CCA	0.734	1
ADA	0.689	2
DDA	0.675	3
DAA	0.653	4
AAA	0.635	5
AAC	0.572	6
BBB	0.567	7
AAB	0.557	8
ABB	0.520	9
CCC	0.493	10
ACC	0.391	11

**Table 16:** Weights of secondary indicators for bone mineral density.

Indicator	Weight	Rank
TMD	0.274	1
BMD	0.266	2
TMC	0.234	3
BMC	0.225	4

**Table 17:** Comprehensive score and ranking of bone mineral density in each treatment group.

Treatment	Comprehensive Score	Rank
CCA	0.729	1
ADA	0.625	2
BBB	0.555	3
AAA	0.538	4
ABB	0.532	5
DAA	0.510	6
ACC	0.408	7
DDA	0.404	8
CCC	0.395	9
AAB	0.379	10
AAC	0.243	11

**Table 18:** Weight of secondary indicators for body measurement.

Indicator	Weight	Rank
RH	0.240	1
CH	0.208	2
RW	0.193	3
CW	0.190	4
BL	0.169	5

**Table 19:** Comprehensive score and ranking of body measurement in each treatment group.

Treatment	Comprehensive Score	Rank
AAA	0.578	1
ABB	0.525	2
AAB	0.516	3
AAC	0.508	4
BBB	0.498	5
DAA	0.488	6
CCC	0.474	7
CCA	0.450	8
ADA	0.441	9
ACC	0.406	10
DDA	0.102	11

**Table 20:** Weight of secondary indicators for energy and nitrogen retention.

Indicator	Weight	Rank
Apparent nitrogen digestibility	0.403	1
Energy deposition rate	0.339	2
Nitrogen deposition rate	0.258	3

**Table 21:** Comprehensive score and ranking of energy and nitrogen retention in each treatment group.

Treatment	Comprehensive Score	Rank
ABB	0.854	1
BBB	0.766	2
DAA	0.764	3
ADA	0.706	4
DDA	0.702	5
CCA	0.666	6
AAB	0.592	7
AAA	0.547	8
ACC	0.419	9
CCC	0.409	10
AAC	0.407	11

**Table 22:** Weight of primary indicators.

Indicator	Weight	Rank
Growth performance	0.162	1
Energy and nitrogen retention	0.150	2
Carcass quality	0.126	3
Trabecular parameters	0.124	4
Bone parameters	0.118	5
Nutrient digestibility	0.117	6
Body measurements	0.104	7
Bone density	0.098	8

**Table 23:** Comprehensive score and ranking in each treatment group.

Treatment	Comprehensive Score	Rank
ABB	0.717	1
AAA	0.690	2
BBB	0.643	3
ADA	0.642	4
DAA	0.627	5
AAB	0.562	6
CCA	0.562	7
AAC	0.445	8
ACC	0.387	9
CCC	0.386	10
DDA	0.359	11

## Data Availability

The data supporting the findings of this study are available from the corresponding author upon reasonable request. The full dataset is not publicly deposited in a public repository due to intellectual property protection associated with the ongoing project.

## Supplementary Materials

The following supporting information can be downloaded at https://www.mdpi.com/article/10.3390/ani16213388/s1.
