# Supplementary material for: Dynamic Nutritional Trajectories in Growing–Finishing Pigs: A CRITIC-Based Evaluation and SHAP-Interpreted Final Body Weight Prediction
Source: Animals (Basel). 2026 Oct 27;16(21):3388. doi: 10.3390/ani16213388 (PMC13762024; doi:10.3390/ani16213388)
Supplement: Supplementary file 1 [file animals-16-03388-s001.zip › animals-4597988-supplementary.pdf]

animals

MDPI

Table S1. Effects of dietary nutrient concentrations on growth performance of growing–finishing pigs.

Treatment	Initial BW	Final BW	Total weight gain	Relative growth rate	ADG	ADFI	FCR
	kg	kg	kg	%	kg	kg	
25-50 kg							
A	29.1	50.7^{a}	21.6^{a}	74.3^{a}	0.718^{a}	1.123	1.563
B	29.3	48.9^{a}	19.6^{b}	67.0^{a}	0.653^{b}	1.113	1.706
C	29.1	45.2^{b}	16.1^{c}	55.4^{b}	0.537^{c}	1.000	1.864
D	29.0	42.0^{c}	13.0^{d}	45.0^{c}	0.433^{d}	0.836	1.924
SEM	0.285	0.915	0.863	0.031	0.029	0.049	0.087
P-value	0.982	0.000	0.000	0.000	0.000	0.128	0.500
50-75 kg							
AA	50.5^{a}	76.8^{ab}	26.3^{a}	52.1^{a}	0.656^{a}	1.871^{ab}	2.851^{c}
BB	48.9^{a}	77.8^{a}	28.9^{a}	59.4^{a}	0.723^{a}	2.188^{a}	3.031^{c}
CC	45.2^{b}	72.5^{bc}	27.7^{a}	60.3^{a}	0.682^{a}	2.216^{a}	3.281^{bc}
DD	41.8^{c}	56.9^{d}	15.1^{d}	35.9^{b}	0.376^{c}	1.393^{c}	3.721^{ab}
AB	50.4^{a}	78.3^{a}	27.9^{a}	55.4^{a}	0.698^{a}	2.038^{a}	2.905^{c}
AC	50.7^{a}	70.7^{c}	20.0^{b}	39.7^{b}	0.502^{b}	1.963^{a}	3.900^{a}
AD	50.8^{a}	68.2^{c}	17.4^{bc}	34.3^{b}	0.436^{bc}	1.415^{bc}	3.246^{bc}
DA	41.9^{c}	65.2^{c}	23.3^{b}	55.6^{a}	0.583^{ab}	1.987^{a}	3.411^{bc}
SEM	0.748	1.572	1.192	0.024	0.030	0.080	0.097
P-value	0.000	0.000	0.000	0.000	0.000	0.002	0.002
75-100 kg							
AAA	76.9^{a}	115.5^{a}	38.6^{a}	50.3^{cd}	0.965^{a}	2.617^{bcd}	2.736^{c}
BBB	77.5^{a}	115.8^{a}	38.3^{a}	49.5^{d}	0.958^{a}	2.780^{ab}	2.911^{c}
CCC	72.7^{b}	101.1^{cde}	28.4^{cd}	39.4^{ef}	0.711^{cd}	2.552^{bcd}	3.595^{a}
ABB	78.8^{a}	117.3^{a}	38.4^{a}	48.5^{d}	0.961^{a}	2.990^{ab}	3.112^{abc}
ACC	70.5^{bc}	97.9^{de}	27.4^{cd}	38.8^{ef}	0.686^{cd}	2.172^{d}	3.170^{abc}
AAB	76.7^{a}	105.5^{bc}	28.8^{cd}	40.3^{e}	0.720^{cd}	2.153^{d}	3.012^{abc}
AAC	76.6^{a}	101.8^{cde}	25.2^{d}	35.2^{f}	0.630^{d}	2.262^{cd}	3.588^{a}
CCA	72.7^{b}	104.4^{bcd}	31.7^{bc}	38.9^{ef}	0.793^{bc}	2.814^{ab}	3.558^{ab}
DAA	65.2^{d}	102.2^{cde}	37.0^{ab}	54.6^{bc}	0.926^{ab}	2.692^{abc}	2.914^{bc}
DDA	57.2^{e}	97.0^{e}	39.8^{a}	64.4^{a}	0.996^{a}	3.026^{ab}	3.037^{abc}
ADA	68.5^{c}	108.6^{b}	40.1^{a}	60.6^{b}	1.002^{a}	3.149^{a}	3.133^{abc}
SEM	0.993	1.220	0.918	0.016	0.023	0.061	0.066
P-value	0.000	0.000	0.000	0.000	0.000	0.000	0.008

Note: In the same row, values with different letter superscripts mean significant difference ($P<0.05$), while with same letter or no letter superscripts mean no significant difference ($P>0.05$). The same as below.

Table S2. Effects of dietary nutrient concentrations on apparent nutrient digestibility of growing–finishing pigs.

Treatment	Fat digestibility	Protein digestibility	Fiber digestibility	Energy digestibility
	%	%	%	%
25-50 kg				
A	91.50[a]	52.17[a]	78.00[b]	87.39[ab]
B	91.68[a]	52.53[a]	88.85[a]	91.98[a]
C	80.48[b]	49.03[ab]	86.45[a]	82.28[b]
D	67.20[c]	40.53[c]	78.13[b]	68.88[c]
SEM	3.056	1.561	2.165	2.489
P-value	0.000	0.000	0.036	0.000
50-75 kg				
AA	89.10[a]	50.33[a]	67.73[a]	90.97[a]
BB	89.87[a]	50.60[a]	68.63[a]	91.63[a]
CC	89.50[a]	44.37[ab]	67.07[a]	87.57[a]
DD	83.93[ab]	44.90[ab]	50.23[b]	75.50[ab]
AB	90.80[a]	50.43[a]	69.80[a]	91.77[a]
AC	89.13[a]	46.87[ab]	71.30[a]	88.70[a]
AD	76.03[b]	37.80[b]	53.07[b]	65.90[b]
SEM	1.315	1.420	4.262	2.512
P-value	0.005	0.010	0.002	0.007
75-100 kg				
AAA	85.70[abc]	48.57[a]	72.10[b]	87.00[ab]
BBB	87.23[abc]	48.27[a]	77.50[ab]	89.30[a]
CCC	80.93[bcd]	46.67[ab]	66.43[b]	84.30[abc]
ABB	87.93[ab]	51.67[a]	85.83[a]	89.90[a]
ACC	78.17[cd]	42.32[ab]	68.51[b]	80.25[bc]
AAB	74.97[d]	35.80[b]	69.20[b]	76.17[c]
AAC	77.00[cd]	35.70[b]	70.57[ab]	77.13[bc]
CCA	85.63[abc]	50.87[a]	69.10[b]	88.50[a]
DAA	92.10[a]	54.50[a]	81.67[ab]	93.10[a]
DDA	89.60[ab]	49.43[a]	68.80[ab]	90.07[a]
ADA	88.70[ab]	51.43[a]	76.97[ab]	90.17[a]
SEM	1.711	1.937	4.969	1.820
P-value	0.008	0.001	0.005	0.006

Note: In the same row, values with different letter superscripts mean significant difference ($P<0.05$), while with same letter or no letter superscripts mean no significant difference ($P>0.05$). The same as below.

Table S3. Effects of dietary nutrient concentrations on carcass quality of growing–finishing pigs.

Treatment	Dressing percentage	Skin thickness	Backfat thickness	Carcass straight length	Carcass diagonal length	Loin eye area
	%	cm	cm	cm	cm	cm²
AAA	73.1[ab]	0.34[b]	1.88[a]	100.10[a]	112.20[ab]	35.77[abc]
BBB	71.5[abc]	0.34[b]	1.51[abc]	97.67[abc]	111.00[abc]	42.26[ab]
CCC	69.8[cd]	0.36[b]	1.23[bc]	94.00[bcd]	105.33[cde]	43.66[ab]
ABB	73.5[a]	0.40[ab]	1.16[cd]	99.00[ab]	113.33[a]	36.35[abc]
ACC	70.8[bcd]	0.33[b]	0.62[d]	93.67[cd]	105.00[cde]	48.71[a]
AAB	70.6[cd]	0.37[ab]	1.54[abc]	94.67[bcd]	106.33[bcd]	34.28[abc]
AAC	68.5[d]	0.37[ab]	1.31[abc]	93.67[cd]	103.33[de]	42.21[ab]
CCA	70.4[cd]	0.34[b]	1.28[bc]	94.33[bcd]	105.33[cde]	28.35[bc]
DAA	71.5[abc]	0.36[b]	1.81[ab]	97.33[abc]	107.67[abcd]	36.26[abc]
DDA	68.5[d]	0.46[a]	1.01[cd]	91.67[d]	99.67[e]	25.39[c]
ADA	71.9[abc]	0.35[b]	1.51[abc]	94.00[bcd]	105.67[cde]	40.05[abc]
SEM	0.273	0.007	0.055	0.646	0.806	1.593
P-value	0.000	0.025	0.000	0.000	0.000	0.035

Note: In the same row, values with different letter superscripts mean significant difference (P＜0.05), while with same letter or no letter superscripts mean no significant difference (P＞0.05). The same as below.

Table S4. Effect of experimental dietary nutrients content on carcass bone volume & area.

Treatment	BV	TV	BV/TV	BS	BS/TV	BS/BV
	mm³	mm³		mm²	mm⁻¹	mm⁻¹
25-50kg						
A	486.8	1273.7	0.379	8229.3	6.435	17.226
B	498.8	1415.1	0.351	8176.0	5.758	16.515
C	431.3	1234.2	0.349	7278.1	5.884	17.222
D	416.4	1295.0	0.323	6936.9	5.372	16.744
SEM	23.716	34.079	0.015	298.760	0.159	0.381
P-value	0.578	0.286	0.646	0.342	0.113	0.906
50-75kg						
AA	630.9	1637.8	0.387	9626.6	5.876	15.313
BB	589.3	1609.4	0.362	8891.0	5.464	15.103
CC	726.4	1681.2	0.449	10717.0	6.428	14.901
DD	462.5	1350.8	0.341	7239.1	5.341	15.652
AB	685.5	1518.8	0.450	9400.1	6.189	13.785
AC	574.1	1578.4	0.369	9005.8	5.776	15.702
AD	612.1	1688.3	0.363	9078.4	5.384	14.845
SEM	23.622	44.976	0.014	324.768	0.126	0.280
P-value	0.068	0.511	0.258	0.190	0.148	0.677
75-100kg						
AAA	762.6	1919.1[b]	0.398	10874.3[abc]	5.679	14.390
BBB	730.2	1872.8[b]	0.389	10096.2[bc]	5.372	13.813
CCC	668.5	1882.1[b]	0.355	10480.0[abc]	5.564	15.830
ABB	867.0	2425.9[a]	0.357	13062.5[a]	5.370	15.079
ACC	618.0	1752.6[bc]	0.355	8802.5[bc]	4.991	14.122
AAB	718.3	1955.0[b]	0.365	11110.5[ab]	5.668	15.692
AAC	694.0	1797.1[bc]	0.384	10528.8[abc]	5.835	15.323
CCA	824.1	1871.4[b]	0.442	11078.4[ab]	5.946	13.461
DAA	757.9	1888.1[b]	0.402	11103.8[ab]	5.908	14.649
DDA	578.2	1512.6[c]	0.380	8090.1[c]	5.319	14.019
ADA	754.4	1813.6[bc]	0.415	10345.9[abc]	5.701	13.998
SEM	19.247	36.766	0.007	255.631	0.079	0.204
P-value	0.162	0.001	0.452	0.026	0.455	0.317

Note: In the same row, values with different letter superscripts mean significant difference (P＜0.05), while with same letter or no letter superscripts mean no significant difference (P＞0.05). The same as below.

Table S5. Effects of dietary nutrient concentration on bone histomorphometry in growing–finishing pigs.

Treatment	Tb.Th	Tb.Sp	Tb.N	Tb.Pf	SMI
	mm	mm	mm^{-1}	mm^{-1}	
25-50kg					
A	0.18	0.34^{b}	1.92^{a}	-1.16	0.41
B	0.19	0.40^{ab}	1.71^{ab}	0.04	0.63
C	0.19	0.38^{ab}	1.78^{a}	0.16	0.54
D	0.19	0.48^{a}	1.51^{b}	0.61	0.71
SEM	0.004	0.019	0.049	0.526	0.065
P-value	0.914	0.044	0.012	0.714	0.439
50-75kg					
AA	0.20	0.43	1.60	-1.42	0.29
BB	0.21	0.60	1.36	-1.37	0.32
CC	0.21	0.34	1.83	-3.77	-0.06
DD	0.20	0.56	1.32	-0.84	0.49
AB	0.22	0.43	1.55	-3.95	-0.08
AC	0.20	0.58	1.45	-1.59	0.33
AD	0.21	0.75	1.06	-1.81	0.36
SEM	0.003	0.045	0.071	0.467	0.086
P-value	0.699	0.250	0.119	0.483	0.561
75-100kg					
AAA	0.22	0.49^{b}	1.46	-2.20	0.17
BBB	0.22	0.65^{ab}	1.20	-2.08	0.19
CCC	0.20	0.51^{b}	1.45	-0.94	0.42
ABB	0.21	0.51^{b}	1.42	-0.83	0.46
ACC	0.22	0.93^{a}	0.93	-1.50	0.34
AAB	0.20	0.43^{b}	1.60	-0.88	0.42
AAC	0.20	0.49^{b}	1.47	-1.78	0.24
CCA	0.23	0.43^{b}	1.54	-3.22	0.07
DAA	0.21	0.43^{b}	1.63	-1.94	0.31
DDA	0.23	0.65^{ab}	1.24	-1.87	0.28
ADA	0.22	0.46^{b}	1.50	-2.62	0.09
SEM	0.003	0.031	0.046	0.215	0.038
P-value	0.343	0.038	0.093	0.572	0.491

Note: In the same row, values with different letter superscripts mean significant difference ($P<0.05$), while with same letter or no letter superscripts mean no significant difference ($P>0.05$). The same as below.

Table S6. Effect of experimental dietary nutrients content on bone mineral density.

Treatment	BMD mg/cm³	BMC mg	TMD mg/cm³	TMC mg
25-50kg				
A	181.2	232.7	354.2	174.7
B	175.2	248.7	370.0	184.9
C	167.5	207.5	361.3	158.1
D	161.0	207.9	372.9	155.9
SEM	7.386	12.057	5.665	10.236
P-value	0.820	0.597	0.682	0.754
50-75kg				
AA	183.1	297.9	364.6	230.0
BB	180.1	292.6	387.2	227.5
CC	214.0	347.0	377.5	275.3
DD	169.5	229.7	381.5	176.6
AB	223.7	341.7	398.0	274.0
AC	162.6	247.2	343.7	197.2
AD	182.2	307.6	392.2	240.3
SEM	7.958	12.860	6.798	10.550
P-value	0.384	0.115	0.430	0.102
75-100kg				
AAA	193.1	370.9	389.3[ab]	298.7
BBB	195.4	366.0	402.8[ab]	293.2
CCC	172.6	324.5	373.4[abc]	251.1
ABB	170.5	414.4	380.2[abc]	330.1
ACC	177.4	308.7	388.5[ab]	240.0
AAB	166.9	329.0	362.2[bc]	262.6
AAC	155.5	278.4	332.2[c]	226.7
CCA	226.7	421.4	417.9[a]	343.4
DAA	193.4	364.8	379.8[abc]	287.2
DDA	186.8	284.2	391.6[ab]	226.7
ADA	213.2	387.2	405.4[ab]	307.6
SEM	4.497	10.532	4.547	8.856
P-value	0.084	0.084	0.015	0.097

Note: In the same row, values with different letter superscripts mean significant difference ($P < 0.05$), while with same letter or no letter superscripts mean no significant difference ($P > 0.05$). The same as below.

Table S7. End-of-stage body conformation trait data of pigs in each group.

Treatment	BW Kg	BL cm	RH cm	RW cm	CH cm	CW cm
25-50kg						
A	50.7^{a}	81.5	53.5	25.5	50.8	24.8
B	48.9^{a}	80.3	54.5	24.3	52.3	24.3
C	45.2^{b}	81.0	53.0	24.0	51.5	23.8
D	42.0^{c}	76.5	52.3	23.8	50.3	23.3
SEM	0.915	0.828	0.463	0.287	0.467	0.224
P-value	0.000	0.119	0.406	0.127	0.493	0.082
50-75kg						
AA	76.8^{ab}	92.8^{ab}	60.5^{a}	31.0^{a}	59.0^{a}	30.3^{a}
BB	77.8^{a}	92.7^{ab}	61.0^{a}	29.3^{ab}	59.0^{a}	28.7^{ab}
CC	72.5^{bc}	91.0^{ab}	60.7^{a}	29.0^{b}	58.3^{a}	28.3^{ab}
DD	56.9^{d}	80.7^{c}	56.0^{b}	26.7^{c}	53.7^{b}	26.0^{c}
AB	78.3^{a}	94.0^{a}	60.7^{a}	30.0^{ab}	58.7^{a}	30.0^{a}
AC	70.7^{c}	90.0^{ab}	60.0^{a}	29.0^{b}	58.7^{a}	28.0^{b}
AD	68.2^{c}	88.7^{b}	60.0^{a}	29.3^{ab}	57.7^{a}	27.3^{bc}
SEM	1.572	1.002	0.380	0.317	0.479	0.353
P-value	0.000	0.000	0.000	0.001	0.011	0.001
75-100kg						
AAA	115.5^{a}	106.0^{a}	71.0^{ab}	32.5^{ab}	67.2	32.9^{a}
BBB	115.8^{a}	103.8^{abc}	70.3^{ab}	31.5^{abc}	67.2	31.3^{abc}
CCC	101.1^{cde}	95.7^{de}	69.3^{ab}	30.7^{cd}	65.7	30.7^{bc}
ABB	117.3^{a}	105.7^{ab}	73.0^{a}	33.0^{a}	69.7	32.3^{ab}
ACC	97.9^{de}	99.0^{cde}	67.5^{bc}	30.0^{cd}	64.5	30.5^{bc}
AAB	105.5^{bc}	100.7^{abcde}	68.3^{bc}	31.7^{abc}	65.0	31.0^{abc}
AAC	101.8^{cde}	97.3^{de}	68.7^{b}	30.3^{cd}	67.3	31.0^{abc}
CCA	104.4^{bc}	101.3^{abcd}	68.3^{bc}	31.0^{bcd}	63.7	32.0^{abc}
DAA	102.2^{cd}	99.3^{cde}	69.3^{ab}	30.0^{cd}	67.0	30.0^{c}
DDA	97.0^{e}	95.3^{e}	64.7^{c}	29.3^{d}	64.0	30.0^{c}
ADA	108.6^{b}	100.0^{bcde}	69.0^{b}	31.7^{abc}	64.7	31.7^{abc}
SEM	1.220	0.709	0.401	0.207	0.408	0.218
P-value	0.000	0.000	0.002	0.000	0.082	0.006

Note: In the same row, values with different letter superscripts mean significant difference ($P < 0.05$), while with same letter or no letter superscripts mean no significant difference ($P > 0.05$). The same as below.

Table S8. Energy and nitrogen retention in pigs under different nutritional trajectories.

Treatment	Energy deposition rate	Apparent nitrogen digestibility	Nitrogen deposition rate
	%	%	%
25-50 kg			
A	13.15^{ab}	91.51^{a}	77.11^{ab}
B	16.24^{a}	94.68^{a}	79.30^{a}
C	9.22^{b}	80.48^{b}	61.49^{b}
D	2.91^{c}	67.18^{c}	37.80^{c}
SEM	1.534	3.058	4.908
P-value	0.001	0.000	0.000
50-75 kg			
AA	23.82^{ab}	89.11^{a}	54.40^{bc}
BB	29.25^{a}	89.88^{a}	51.38^{bc}
AB	33.99^{a}	90.83^{a}	66.21^{ab}
CC	12.78^{ab}	89.51^{a}	54.61^{bc}
AC	34.14^{a}	89.13^{a}	79.20^{a}
DD	33.73^{a}	83.94^{ab}	60.59^{abc}
AD	6.95^{b}	76.03b	42.79^{c}
SEM	2.727	1.317	3.099
P-value	0.004	0.005	0.021
75-100 kg			
AAA	32.05^{b}	85.69^{abc}	40.38
BBB	53.32^{ab}	87.24^{abc}	64.49
ABB	39.46^{ab}	87.90^{abc}	74.32
AAB	59.22^{a}	80.96^{abc}	56.28
CCC	31.42^{b}	74.98^{c}	31.07
ACC	38.21^{ab}	75.28^{c}	43.85
AAC	31.09^{b}	76.99^{bc}	37.19
CCA	51.12^{ab}	85.64^{abc}	48.53
DAA	46.76^{ab}	92.10^{a}	52.66
DDA	44.33^{ab}	89.60^{ab}	49.60
ADA	42.97^{ab}	88.69^{abc}	47.79
SEM	2.138	1.250	3.576
P-value	0.026	0.008	0.283

Note: In the same row, values with different letter superscripts mean significant difference ($P<0.05$), while with same letter or no letter superscripts mean no significant difference ($P>0.05$). The same as below.

Table S9. Weighted contribution of backfat thickness to the comprehensive scores.

Treatment	Backfat (cm)	Standardized Score	Weighted Contribution	Original Rank
AAA	1.88	0.000	0.000	1
ABB	1.16	0.571	0.114	2
AAB	1.54	0.270	0.054	3
ADA	1.51	0.294	0.059	4
BBB	1.51	0.294	0.059	5
AAC	1.31	0.452	0.090	6
ACC	0.62	1.000	0.200	7
DAA	1.81	0.056	0.011	8
CCC	1.23	0.516	0.103	9
CCA	1.28	0.476	0.095	10
DDA	1.01	0.690	0.138	11

Table S10. Ranking stability comparison without Backfat Thickness.

Indicator	Original Weight	Re-Normalized Weight (Excluding Backfat)
Loin eye area	0.243	0.304
Dressing percentage	0.155	0.194
Carcass straight length	0.140	0.175
Skin thickness	0.135	0.169
Carcass diagonal length	0.126	0.158

Table S11. The equal-weight robustness of growth performance in each treatment group.

Treatment	CRITIC score	Equal-weight score	Difference	Relative deviation	CRITIC rank	Equal-weight rank	Rank change
AAA	0.885	0.862	−0.023	−2.6%	1	1	0
ABB	0.758	0.741	−0.017	−2.2%	2	2	0
AAB	0.643	0.655	0.012	1.90%	3	3	0
ADA	0.613	0.608	−0.005	−0.8%	4	4	0
BBB	0.54	0.522	−0.018	−3.3%	5	5	0
AAC	0.471	0.485	0.014	3.00%	6	6	0
ACC	0.313	0.334	+0.021	+6.7%	7	7	0
DAA	0.312	0.309	−0.003	−1.0%	8	8	0
CCC	0.155	0.172	+0.017	+11.0%	9	9	0
CCA	0.149	0.151	0.002	1.30%	10	10	0
DDA	0.149	0.148	−0.001	−0.7%	10	11	−1

Note: Spearman $\varrho = 0.9977$.

Table S12. The equal-weight robustness of nutrient digestibility in each treatment group.

Treatment	CRITIC score	Equal-weight score	Difference	Relative deviation	CRITIC rank	Equal-weight rank	Rank change
DAA	0.925	0.914	−0.0108	−1.17	1	1	0
ABB	0.881	0.865	−0.0164	−1.86	2	2	0
ADA	0.834	0.812	−0.0225	−2.70	3	3	0
BBB	0.801	0.775	−0.0265	−3.30	4	4	0
DDA	0.779	0.751	−0.0284	−3.64	5	5	0
CCA	0.749	0.719	−0.0300	−4.01	6	6	0
AAA	0.685	0.654	−0.0305	−4.46	7	7	0
AAB	0.646	0.618	−0.0280	−4.33	8	8	0
ACC	0.587	0.566	−0.0205	−3.50	9	9	0
AAC	0.351	0.378	0.0275	7.83	10	10	0
CCC	0.322	0.351	0.0289	8.96	11	11	0

Note: Spearman ρ = 0.977.

Table S13. The equal-weight robustness of carcass quality in each treatment group.

Treatment	CRITIC score	Equal-weight score	Difference	Relative deviation	CRITIC rank	Equal-weight rank	Rank change
AAA	0.654	0.6443	-0.0097	-1.4793	1	1	0
BBB	0.602	0.5951	-0.0069	-1.1498	2	2	0
ABB	0.587	0.5811	-0.0059	-1.0017	3	3	0
DAA	0.558	0.554	-0.004	-0.7186	4	4	0
ADA	0.529	0.5272	-0.0018	-0.3341	5	5	0
ACC	0.488	0.4895	0.0015	0.3154	6	6	0
CCC	0.478	0.4802	0.0022	0.4523	7	7	0
AAB	0.47	0.4728	0.0028	0.5972	8	8	0
AAC	0.432	0.4375	0.0055	1.2751	9	9	0
CCA	0.403	0.4105	0.0075	1.8596	10	10	0
DDA	0.225	0.2369	0.0119	5.2932	11	11	0

Note: Spearman ρ = 0.990.

Table S14. The equal-weight robustness of bone parameters in each treatment group.

Treatment	CRITIC score	Equal-weight score	Difference	Relative deviation	CRITIC rank	Equal-weight rank	Rank change
ABB	0.574	0.5622	-0.0118	-2.0539	1	1	0
DAA	0.535	0.5294	-0.0056	-1.0559	2	2	0
CCA	0.531	0.526	-0.005	-0.9504	3	3	0
AAB	0.527	0.5225	-0.0045	-0.8599	4	4	0
AAC	0.513	0.5107	-0.0023	-0.4393	5	5	0
AAA	0.499	0.4989	-0.0001	-0.0115	6	6	0
CCC	0.487	0.4892	0.0022	0.4579	7	7	0
ADA	0.481	0.4843	0.0033	0.679	8	8	0
BBB	0.414	0.4271	0.0131	3.166	9	9	0
DDA	0.31	0.3341	0.0241	7.784	10	10	0
ACC	0.306	0.3302	0.0242	7.8952	11	11	0

Note: Spearman ρ = 0.989.

Table S15. The equal-weight robustness of trabecular bone in each treatment group.

Treatment	CRITIC score	Equal-weight score	Difference	Relative deviation	CRITIC rank	Equal-weight rank	Rank change
CCA	0.734	0.7195	-0.0145	-1.9775	1	1	0
ADA	0.689	0.6761	-0.0129	-1.8715	2	2	0
DDA	0.675	0.6629	-0.0121	-1.7938	3	3	0
DAA	0.653	0.6421	-0.0109	-1.6719	4	4	0
AAA	0.635	0.6251	-0.0099	-1.5549	5	5	0
AAC	0.572	0.5669	-0.0051	-0.8977	6	6	0
BBB	0.567	0.5621	-0.0049	-0.8707	7	7	0
AAB	0.557	0.5529	-0.0041	-0.7376	8	8	0
ABB	0.52	0.5189	-0.0011	-0.2049	9	9	0
CCC	0.493	0.494	0.001	0.1971	10	10	0
ACC	0.391	0.3998	0.0088	2.249	11	11	0

Note: Spearman ρ = 0.990.

Table S16. The equal-weight robustness of bone mineral density in each treatment group.

Treatment	CRITIC score	Equal-weight score	Difference	Relative deviation	CRITIC rank	Equal-weight rank	Rank change
CCA	0.729	0.728	-0.001	-0.1414	1	1	0
ADA	0.625	0.6245	-0.0005	-0.0798	2	2	0
BBB	0.555	0.5551	0.0001	0.0097	3	3	0
AAA	0.538	0.5381	0.0001	0.0104	4	4	0
ABB	0.532	0.5322	0.0002	0.0323	5	5	0
DAA	0.51	0.5104	0.0004	0.0834	6	6	0
ACC	0.408	0.4092	0.0012	0.2886	7	7	0
DDA	0.404	0.4053	0.0013	0.3192	8	8	0
CCC	0.395	0.3963	0.0013	0.3327	9	9	0
AAB	0.379	0.3804	0.0014	0.3823	10	10	0
AAC	0.243	0.2449	0.0019	0.7698	11	11	0

Note: Spearman ρ = 0.995.

Table S17. The equal-weight robustness of body measurement in each treatment group.

Treatment	CRITIC score	Equal-weight score	Difference	Relative deviation	CRITIC rank	Equal-weight rank	Rank change
AAA	0.578	0.5766	-0.0014	-0.2437	1	1	0
ABB	0.525	0.5245	-0.0005	-0.0965	2	2	0
AAB	0.516	0.5157	-0.0003	-0.0524	3	3	0
AAC	0.508	0.5078	-0.0002	-0.0308	4	4	0
BBB	0.498	0.4981	0.0001	0.0148	5	5	0
DAA	0.488	0.4882	0.0002	0.0311	6	6	0
CCC	0.474	0.4744	0.0004	0.0941	7	7	0
CCA	0.45	0.451	0.001	0.2124	8	8	0
ADA	0.441	0.4421	0.0011	0.2593	9	9	0
ACC	0.406	0.4076	0.0016	0.4027	10	10	0
DDA	0.102	0.1033	0.0013	1.3176	11	11	0

Note: Spearman ρ = 0.997.

Table S18. The equal-weight robustness of energy and nitrogen retention in each treatment group.

Treatment	CRITIC score	Equal-weight score	Difference	Relative deviation	CRITIC rank	Equal-weight rank	Rank change
ABB	0.854	0.849	-0.005	-0.5875	1	1	0
BBB	0.766	0.758	-0.008	-1.0401	2	2	0
DAA	0.764	0.7559	-0.0081	-1.0604	3	3	0
ADA	0.706	0.697	-0.009	-1.2698	4	4	0
DDA	0.702	0.6931	-0.0089	-1.2701	5	5	0
CCA	0.666	0.6583	-0.0077	-1.1593	6	6	0
AAB	0.592	0.5882	-0.0038	-0.6442	7	7	0
AAA	0.547	0.5453	-0.0017	-0.3065	8	8	0
ACC	0.419	0.422	0.003	0.7203	9	9	0
CCC	0.409	0.4129	0.0039	0.9488	10	10	0
AAC	0.407	0.4106	0.0036	0.8742	11	11	0

Note: Spearman ϱ = 0.999.
